# Intraparticle Double‐Scattering‐Decoded Sonogenetics for Augmenting Immune Checkpoint Blockade and CAR‐T Therapy

**DOI:** 10.1002/advs.202203106

**Published:** 2022-09-25

**Authors:** Duo Wang, Mengqi Zhang, Yan Zhang, Guanhua Qiu, Jie Chen, Xiaoqi Zhu, Cunqing Kong, Xiuxin Lu, Xiayi Liang, Lixia Duan, Chao Fang, Junjie Liu, Kun Zhang, Tao Luo

**Affiliations:** ^1^ Department of Medical Ultrasound Department of Interventional Therapy and Department of Gastrointestinal Surgery Guangxi Medical University Cancer Hospital Guangxi Medical University No. 71 Hedi Road Nanning 530021 P. R. China; ^2^ Central Laboratory and Ultrasound Research and Education Institute Shanghai Tenth People's Hospital Tongji University School of Medicine No. 301 Yan‐chang‐zhong Road Shanghai 200072 P. R. China; ^3^ National Center for International Research of Bio‐targeting Theranostics Guangxi Key Laboratory of Bio‐targeting Theranostics Collaborative Innovation Center for Targeting Tumor Diagnosis and Therapy Guangxi Medical University No. 22 Shuangyong Road Nanning 530021 P. R. China

**Keywords:** chimeric antigen receptor‐T replication, chimeric antigen receptor‐T trafficking and persistence, immunosuppressive tumor microenvironment, intraparticle double‐scattering, sonogenetics, vascular normalization

## Abstract

Genetically arming new chimeric antigen receptors (CARs) on T cells is a prevalent method to fulfill CAR‐T immunotherapy. However, this approach fails to completely address the poor infiltration, complex immunosuppressive tumor microenvironment (ITM), and insufficient immune cells, which are recognized as the three dominant hurdles to discouraging the trafficking and persistence of CAR‐T and immune checkpoint blockade (ICB) immunotherapies against solid tumors. To address the three hurdles, a sonoimmunity‐engineered nanoplatform is designed in which a rattle‐type‐structured carrier enables intraparticle‐double‐scattering to generate massive reactive oxygen species (ROS) during the sonodynamic process. Abundant ROS accumulation can directly kill tumor cells, release antigens, and activate systematic immune responses for expanding effector T or CAR‐T cells, while alleviating ITM via immunosuppressive macrophage polarization and reduction in pro‐tumorigenic cytokine secretion. Furthermore, the co‐loaded phosphodiesterase‐5 inhibitors release nitric oxide (NO) to impel vascular normalization and open the infiltration barrier (IB) for allowing more T cells to enter into the tumor. Systematic experiments demonstrate the feasibility of such intraparticle‐double‐scattering‐decoded sonogenetics in the sonoimmunity‐engineered nanoplatforms for expanding effector T or CAR‐T cells, thereby promoting their infiltration into tumors and alleviating ITM. These compelling actions lead to excellent CAR‐T and ICB immunotherapies against solid tumors with repressed tumor metastasis.

## Graphical abstract

An intraparticle double‐scattering‐decoded sonogenetics has been developed to promote reactive oxygen species production, activate systematic immune responses, mitigate immunosuppressive tumor microenvironment, remodel vascular homeostasis (or propel vascular normalization), open infiltration barrier, which cooperatively realizes the persistence and normal function, expedite the infiltration, replication or expansion of chimeric antigen receptor (CAR)‐T cells to potentiate the CAR‐T immunotherapy against solid tumor.

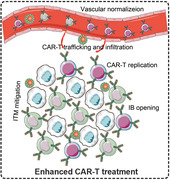

## 1 Introduction

Adoptive T cells that are genetically engineered with chimeric antigen receptor (CAR) have emerged as a promising method for conducting immunotherapy after immune checkpoint blockade (ICB) therapy.^[^ ^1^ ^]^ Despite attracting increasing attention, CAR‐T immunotherapy only benefits hematologic malignancies; furthermore, solid tumors proved refractory to CAR‐T immunotherapy in numerous clinical cases,^[^ ^2^, ^3^ ^]^ where patients received no evident response and clinical benefits.^[^ ^4^ ^]^ Akin to ICB, the disappointing result can be attributed to the low trafficking efficiency and non‐persistence of CAR‐T cells, which are traits that complex immunosuppressive tumor microenvironment (ITM), insufficient CAR‐T cells, and poor CAR‐T infiltrations bring about.^[^ ^5^, ^6^, ^7^ ^]^ Consequently, great efforts and advances have been made to reinforce CAR‐T immunotherapy and ICB.^[^ ^5^, ^6^, ^7^, ^8^, ^9^ ^]^ In an attempt to overcome ITM, engineering various specific CARs that can bind to corresponding tumor antigens has been identified as a general method to mitigate ITM,^[^ ^10^, ^11^, ^12^, ^13^ ^]^ which also serves to promote CAR‐T cell trafficking and effector persistence.^[^ ^14^, ^15^ ^]^ However, anchoring new CARs partly affects ITM and fails to fundamentally and completely eradicate ITM obstacles due to the complexity of ITM (various immunosuppressive cytokines and cells), even when other immunotherapies (e.g., ICB)were combined.^[^ ^16^, ^17^, ^18^, ^19^ ^]^ To circumvent poor infiltration, anchoring new CARs disables the breaking of infiltration barriers (IB) associated with hypoxia, tumor burden, vascular abnormity or decrease, and the dense matrix that remains intact and disfavors CAR‐T trafficking, migration, and infiltration.^[^ ^14^, ^15^ ^]^ Despite circumventing intratumoral IB, the dominantly‐used local delivery of CAR‐T cells by using hydrogel as a reservoir is incapable of adequately activating systematic immune responses,^[^ ^20^ ^]^ thereby leading to failure in treating multiple nodules and metastasized ones.^[^ ^21^, ^22^, ^23^ ^]^ Additionally, genetically engineering T cells with new CARs remains ineffective for addressing different concerns (i.e., insufficient CAR‐T in vivo) since the re‐injected CAR‐T cells fail to continually expand, thus highlighting the non‐persistence of CAR‐T immunotherapy for solid tumors.

**Scheme 1:**
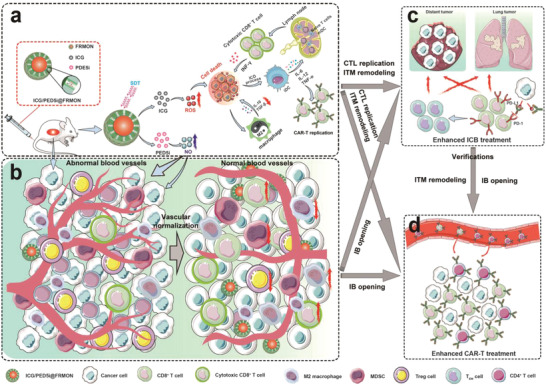
Design principles of intraparticle double‐scattering‐decoded sonogenetics for potentiating immune checkpoint blockading therapy (ICB) (c) and CAR‐T immunotherapy against solid tumor (d) via enhancing systematic immune responses (a), reshaping ITM (a), promoting CAR‐T expansion (d), and remodeling vascular homeostasis (i.e., vascular normalization) (b). In detail, such rattle‐type‐structured sonoimmunity‐engineered nanoplatforms can generate double back‐scattering of ultrasound waves, and elevate ROS production under ICG‐mediated sonocatalysis to directly kill tumor cells, activate systematic immune responses, propel the infiltration and replication of effector T and CAR‐T cells, alleviate ITM including pro‐tumorigenic cytokine secretion decline and pro‐tumorigenic M2‐type macrophage polarization into M1 ones, thus addressing the non‐persistence of CAR‐T and ICB immunotherapies. Intriguingly, entrapped PDE5 inhibitor could produce NO to reshape intertumoral vascular homeostasis and facilitate IB opening, which enables vascular normalization to allow more effector T and CAR‐T cells’ infiltration into solid tumors, addressing the low trafficking efficiency of CAR‐T and ICB immunotherapies. As well, the enhanced ICB immunotherapy indirectly verifies the successful CAR‐T replication, ITM remodeling, and IB opening, benefiting the enhanced CAR‐T immunotherapy.

To completely address these hurdles, we established an intraparticle double‐scattering‐decoded sonogenetic technology to remodel ITM and vascular homeostasis, allowing vascular normalization and CAR‐T or effector T cell expansions to successfully implement Car‐T immunotherapy and ICB against solid tumors (**Scheme** **1**). Differing from ultrasound (US)‐mediated heat‐triggered sonogenetics that is based on optogenetics,^[^ ^24^, ^25^ ^]^ direct ultrasound‐triggered genetic regulation is accessible and can remodel tumor microenvironment and facilitate tumor recession. Fluorinated rattle‐type mesoporous organosilica nanoparticles (FRMONs) featuring intraparticle‐double‐scattering on its two scattering interfaces were obtained and showed higher ultrasound utilization efficiency compared to those of hollow or solid ones with only one scattering interface.^[^ ^26^, ^27^ ^]^ More reactive oxygen species (ROS) were produced in the indocyanine green (ICG)‐mediated sonodynamic process. In addition to directly killing tumor cells and enhancing immunogenic cell death (ICD), ROS is expected to remodel ITM via immunosuppressive M2‐type macrophage polarization into anti‐tumor M1‐type macrophages and a decline in pro‐tumorigenic cytokine secretion (Scheme 1a).^[^ ^21^, ^28^, ^29^ ^]^ ITM overturning could unlock the ITM‐arising imprisonment toward effector T or CAR‐T cells, addressing the non‐persistence of ICB or CAR‐T immunotherapy (Scheme 1c,d).

Entrapped phosphodiesterase‐5 inhibitors (PDE5i) can produce NO to reshape intratumoral vascular homeostasis,^[^ ^30^, ^31^ ^]^ which enables vascular normalization to allow more effector T or CAR‐T cells into the tumors (Scheme 1b);^[^ ^32^, ^33^, ^34^ ^]^ thus, this leads to the low trafficking efficiency of CAR‐T cells. More significantly, direct killing and ICD could release rich antigens for stimulating antigen‐presenting cells (APCs) to release cytokines.^[^ ^29^ ^]^ The cytokines propel the replication and propagation of CD8+ T lymphocytes (CTLs) or CAR‐T cells, thus supplying adequate ammunition to address the poor persistence of CAR‐T immunotherapy and ICB (Scheme 1c,d). Systematic results validated that such intraparticle double‐scattering‐decoded sonogenetics in ICG/PDE5i@FRMON reactivated the exhausted immune responses, elevated the immune potency, remodeled ITM, propelled vascular normalization, opened IB, and induced an influx of endogenous tumor‐specific CD8(+) T cells. All these appealing actions contributed to the boosted ICD and ICB with repressed primary and distant tumor progression and reduced metastasis in vitro and in vivo The success of ICD and ICB immunotherapy indirectly verified the successful ITM, vascular homeostasis re‐shaping, and systematic immune response activation, thereby ensuring massive replication of NKG2D CARs‐engineered CAR‐T cells for exerting the persistent CAR‐T immunotherapy against solid MDA‐MB‐231 tumor in NSG mice in vivo. Collectively, the unprecedented intraparticle‐double‐scattering‐encoded sonogenetic modulation in such sonoimmunity‐engineered nanoplatforms addressed the three bottlenecks of CAR‐T immunotherapy and ICB, thus functioning as a general method to implement CAR‐T and ICB immunotherapies against solid tumors.

## 2 Results

### 2.1 Sonoimmunity‐Engineered Nanoplatforms (ICG/PDE5i@FRMON) Synthesis

**Figure 1:**
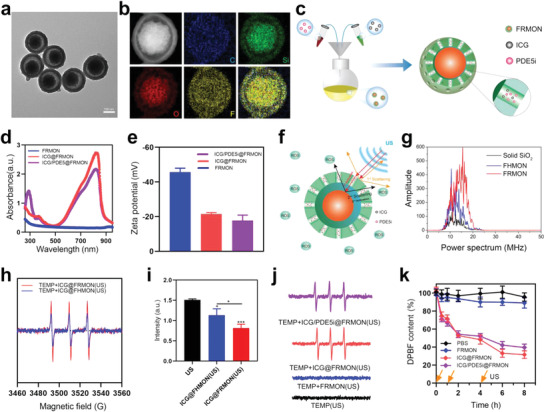
Synthesis and intraparticle‐double‐scattering tests of such rattle‐type‐structured sonoimmunity‐engineered nanoplatforms (ICG/PDE5i@FRMON). a,b) TEM (a) and TEM‐rooted atom mapping (b) images of FRMON carriers, where F atoms are uniformly distributed in nanoparticles. c) Synthesis schematic of such rattle‐type‐structured sonoimmunity‐engineered nanoplatforms (ICG/PDE5i@FRMON). d,e) UV–vis spectra (d) and surface zeta potentials (e) of ICG/PDE5i@FRMON and other two counterparts (FRMON and PDE5i@FRMON). f) Schematic illustration on intraparticle‐double‐scattering in FRMON carriers of ICG/PDE5i@FRMON. g) Backscattering acoustic spectra harvested from solid (SiO_2_), hollow (FHMON), and rattle‐type (FRMON) suspensions with identical concentration (0.1 mg mL^−1^), respectively, where a transducer with the frequency of 6–12 MHz was used. h) Electron spin resonance (ESR) spectra of 2,2,6, 6‐tetramethylpiperidine (TEMP)‐captured singlet oxygen emitted from ICG@FRMON and ICG@FHMON in the presence of US irradiation, respectively. i) UV–vis characteristic absorbance intensities of DPBF after different treatments with US alone, ICG@FHMON(US) and ICG@FRMON(US). j) ESR spectra of TEMP‐captured singlet oxygen emitted from different treatments, that is, TEMP(US), TEMP+FRMON(US), TEMP+ICG@FRMON(US) and TEMP+ICG/PDE5i@FRMON(US). k) UV–vis characteristic absorbance intensities of DPBF as a function of time during different treatments with PBS, FRMON, ICG@FRMON(US), and ICG/PDE5i@FRMON, respectively, where US irradiations were implemented at 0, 1, and 4 h, respectively. Data are expressed as mean ± standard deviation (SD) (*n* = 3). Statistical significance between the groups was determined by one‐way analysis of variance (ANOVA), and *P<0.05.

Monodispersed FRMON carriers with a diameter of 200 nm were obtained (**Figure** **1**a,b)^[^ ^35^, ^36^, ^37^ ^]^ wherein fluorine atoms were uniformly distributed (Figure 1b), thus paving a solid foundation for endosomal escape. Larger surface areas with double‐scale pores (4 and 11 nm) are accessible (Figure S1a, Supporting Information), which demonstrates that FRMON can serve as containers to co‐load ICG and PDE5i via electrostatic interaction‐mediated adsorption (Figure 1c). Typical characteristic peaks of ICG and PDE5i corresponding to 780 and 290 nm, respectively, emerge in ICG/PDE5i@FRMON, suggesting the successful co‐loadings of ICG and PDE5i (Figure 1d). No evident structure, size, and morphology variations of FRMONs after co‐loading ICG/PDE5i are found (Figure S1b,c, Supporting Information). Despite failing to vary particle size (Figure S1d, Supporting Information), the entrapments of ICG and PDE5i alter surface zeta potential and vibration strength (Figure 1e and Figure S1e, Supporting Information). The ultimate ICG/PDE5i@FRMON shows high colloidal stability in a phosphate buffer solution (PBS) and fetal bovine serum (FBS) (Figure S1f,g, Supporting Information). According to their dose‐absorbance standard curves (Figure S1h,j, Supporting Information), the loading percentages of ICG and PDE5i in ICG/PDE5i@FRMON are cal. 4.86% and 7.11%, respectively. In addition, the release profiles of ICG and PDE5i from ICG/PDE5i@FRMON display pH‐responsiveness, which means that the specifically acidic tumor microenvironment is more favorable for ICG and PDE5i release (Figure S1i,k, Supporting Information). Akin to previous reports, local ultrasound irradiation can further accelerate their release (Figure S1i,k, Supporting Information).^[^ ^31^, ^38^ ^]^ The low release rates of ICG and PDE5i in FBS guarantee no leakage of PDE5i and ICG from ICG/PDE5i@FRMON during blood circulation after intravenous injection (Figure S2a, Supporting Information). Notably, such sonoimmunity‐engineered nanoplatforms (ICG/PDE5i@FRMON) show neglectable hemolysis even though the concentration becomes 1000 µg mL^−1^ (Figure S2b, Supporting Information), thus guaranteeing the safety of use.

### 2.2 Intraparticle‐Double‐Scattering Test for Promoting ROS Birth

Intraparticle‐double‐scattering in ICG‐loaded FRMON is depicted in Figure 1f, where two scattering convex interfaces (i.e., the surface of inner core and outer shell) in FRMON carriers that can realize two ultrasound wave back‐scatterings is more preferable.^[^ ^27^, ^36^ ^]^ The second scattering on the surface of the inner core is expected to further activate sonosensitizers (i.e., ICG) to give rise to ROS in comparison to the hollow or solid particles featuring only one scatter interface for acoustic scattering. To verify it, fluorinated hollow mesoporous organosilica nanoparticles (FHMONs) with one scattering interface were obtained as a counterpart for comparison,^[^ ^31^, ^37^ ^]^ and the obtained FHMONs share approximately identical particle size and surface potential with FRMON (Figure S3, Supporting Information). Scattering test revealed that FRMON receives considerably‐augmented backscattering signals in comparison to solid SiO_2_ and FHMON (Figure 1g), denoting the occurrence of two convex interfaces‐arising from double scattering in a single FRMON particle. As a result, the intraparticle‐double‐scattering in FRMON is validated to arm ICG@FRMON with a more robust ability to produce ROS than ICG@FHMON during sonodynamic process, since double scattering can significantly improve acoustic utilization efficiency (Figure 1h). 1,3‐Diphenylisobenzofuran (DPBF) degradation results also revealed that the intraparticle‐double‐scattering elicits more ROS birth to oxidize DPBF in ICG@FRMON(US) (Figure 1i). Intriguingly, PDE5i entrapment in ICG/PDE5i@FRMON fails to impair ICG‐mediated ROS production in comparison to ICG@FRMON in the presence of US irradiation (Figure 1j,k). This result indicates that intraparticle‐double‐scattering‐induced high ROS birth is retained in ICG/PDE5i@FRMON, which will present a series of benefits for high trafficking and persistence of ICD, ICB, or CAR‐T immunotherapy by activating systematic immune responses, mitigating ITM, propelling vascular normalization, and reinforcing CAR‐T or effector T replication and infiltration.

### 2.3 In Vitro ROS, NO, and ICD Tests

**Figure 2:**
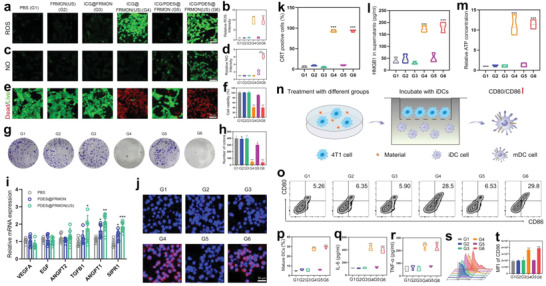
In vitro tests on ROS, NO, apoptosis, vessel normalization, and ICD using such sonoimmunity‐engineered nanoplatforms (ICG/PDE5i@FRMON) for intraparticle‐double‐scattering. a,b) Laser confocal scanning microscopic (LCSM) images (a) and quantitative data (b) of 4T1 cells stained with ROS indicator (i.e., DCFH‐DA) after corresponding treatment in different groups (G1–G6) for evaluating ROS level. c,d) LCSM images (c) and quantitative data (d) of 4T1 cells stained with NO probe (i.e., DAF‐FM DA) after corresponding treatment in different groups (G1–G6) for evaluating NO level. e) LCSM images of 4T1 cells stained with propidium iodide (PI)/calcein assay kit after corresponding treatment in different groups (G1–G6) for evaluating dead (red) and live (green) cells. Data are expressed as mean ± SD (*n* = 4). Statistical significance was determined by ANOVA, and *P<0.05, **P<0.01, and ***P<0.001. f) Cell viability of 4T1 cells after corresponding treatment in different groups (G1–G6) via CCK8 method. g,h) Optical images (g) and the number of 4T1 colonies (h) in colon formation test after 14 days incubation in different groups (G1–G6). Data are expressed as mean ± SD (*n* = 3). Statistical significance was determined by ANOVA, and *P<0.05, **P<0.01, and *P*<0.001. i) Expression levels of mRNA in HUVECs after corresponding treatment in three groups (PBS, PDE5i@FRMON, and PDE5i@FRMON(US)). Data are expressed as mean ± SD (*n* = 6). Statistical significance between the groups was determined by *t*‐test, and *P<0.05, **P<0.01, and *P*<0.001. j,k) LCSM images (j) and CRT‐positive cell percentages (k) of 4T1 cells stained with anti‐CRT antibody after corresponding treatment in different groups (G1–G6), where pink represents CRT. l,m) HMGB1 and ATP levels of 4T1 tumor after corresponding treatment in different groups (G1–G6), which were determined by enzyme‐linked immunosorbent assay (ELISA) and luminometer, respectively. n) Schematic on DC maturation test induced by ICG/PDE5i@FRMON; o,p) Typical flow cytometry (FCM) patterns (o) and quantitative data (p) for determining DCs maturation (CD80+CD86+) after corresponding treatment in different groups (G1–G6). q,r) Expression levels of TNF‐*α* and IL‐6 secreted by matured DCs by ELISA test after corresponding treatment in different groups (G1–G6). s,t) FCM patterns (s) and mean fluorescence intensities (t) of macrophages for determining M1‐type ones (CD86+) after corresponding treatment in different groups (G1–G6). Data are expressed as mean ± SD (*n* = 3). Statistical significance was determined by ANOVA, and *P<0.05, **P<0.01, and ***P<0.001. Note, G1–G6 represent control (PBS), FRMON(US); ICG@FRMON, ICG@FRMON(US), ICG/PDE5i@FRMON and ICG/PDE5i@FRMON(US), respectively; and Scale bar: 50 µm.

Cellular‐level testing revealed that such sonoimmunity‐engineered nanoplatforms unlock intraparticle double‐scattering‐decoded sonogenetics to induce the most robust sonocatalytic processes for producing the most ROS in US and ICG‐contained groups (i.e., ICG@FRMON(US) (G4) and ICG/PDE5i@FRMON(US) (G6)) (**Figure** **2**a,b). PDE5i entrapment equips ICG/PDE5i@FRMON(US) with the most robust ability to generate NO (Figure 2c,d). Notably, the NO level in ICG/PDE5i@FRMON is inferior to ICG/PDE5i@FRMON(US), which can be attributed to the absence of US‐accelerated PDE5i release. G4 and G6 groups, which contributed the maximum number of ROS, are found to induce the most cell deaths and exhibit approximately identical death percentages (Figure 2e,f). Identical results are obtained in the colon formation test, where G4 and G6 exert the most potent inhibition effect to repress the birth of 4T1 colons (Figure 2g,h).

Intriguingly, NO level in ICG/PDE5i@FRMON(US) has not exceeded the threshold where NO‐based anti‐tumor action will be triggered since NO exhibits two‐sided characters (Figure 2e–h),^[^ ^39^, ^40^ ^]^ under which vascular homeostasis remodeling can be attained. As expected, three genes (TGFB1, ANGPT1, and SIPR1) representing vascular maturation of human umbilical vein endothelial cells (HUVECs) are up‐regulated in PDE5i‐involved groups especially when US is applied, while the other three genes (VEGFA, EGF, and ANGPT2) mattering angiogenesis fail to vary (Figure 2i). This phenomenon suggests that PDE5i indeed promoted vascular normalization without interfering with normal blood vessels. Identical results are obtained on another cell lineage (MDA‐MB‐231), where the most ROS and NO births occur to ICG/PDE5i@FRMON(US) treatment (Figure S4, Supporting Information).

Furthermore, in vitro ICD was explored, and some hallmarkers of ICD were inspected. Given that ROS can directly decide ICD, it is expected that such intraparticle double‐scattering‐enhanced ROS birth in US and ICG‐contained groups (G4 and G6) brings about the significant up‐regulation of three ICD hallmarkers including calreticulin (CRT), high mobility group box1protein (HMGB1), and ATP (Figure 2j–m). Additionally, the primary APCs, that is, dendritic cells (DCs), were surveyed (Figure 2n). Depending on the most expressions of CRT, HMGB1, ATP, and G6 results displayed the maximum DC maturation (Figure 2o,p). Consequently, most expressions of IL‐6 and TNF‐*α* secreted by matured DCs are harvested (Figure 2q,r), creating a favorable microenvironment for replication and propagation of CAR‐T cells since IL‐6 and TNF‐*α* could promote T activation and expansion.^[^ ^9^ ^]^ In light of the fact that ROS also directly correlates with ITM,^[^ ^29^ ^]^ G4 and G6 allow the birth of most ROS, thereby effectively promoting macrophage polarization into anti‐tumorigenic M1‐type ones (Figure 2s,t) and successfully mitigating ITM for potentiating CAR‐T immunotherapy against solid tumors.

It is worth noting that fluorocarbon chains in FRMON and induced lysosome rupture permit ICG/PDE5i@FRMON to escape lysosome (Figure S5, Supporting Information), which is in part responsible for the above variations. In detail, fluorocarbon chains would be easily fused with membrane lipids via hydrophobic interactions,^[^ ^21^, ^41^, ^42^ ^]^ thus allowing the rapid diffusion of fluorocarbon chained‐modified nanoparticles across the lipid membranes and inducing lysosome escape within 4 h. As the incubation time exceeds 8 h, the ruptured lysosomes represented by a weak fluorescence signal further favored the escape of nanoparticles, PDE5i, and ICG, which verifies the report.^[^ ^43^ ^]^

### 2.4 In Vivo ICD Activation and Anti‐Tumor Evaluations

**Figure 3:**
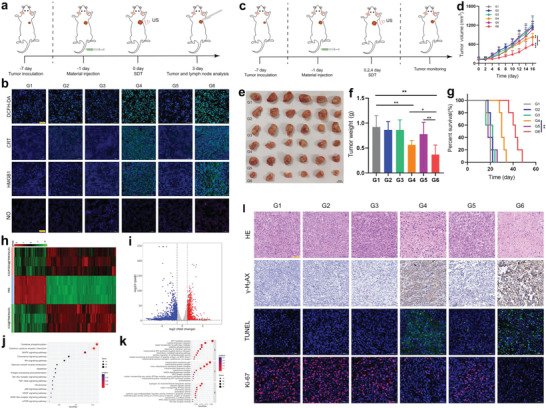
In vivo tests on ICD, mechanistic analysis, and anti‐tumor evaluations using such intraparticle‐double‐scattering‐decoded sonogenetics in ICG/PDE5i@FRMON. a) Operation schematic on in vivo ICD test, where 4T1 tumors and lymph nodes were collected on the 3rd day post‐corresponding treatment in different groups (G1–G6). b) LCSM images of 4T1 tumor slices stained with DCFH‐DA, anti‐CRT antibody, anti‐HMGB1 antibody, and NO fluorescence probe for monitoring the levels of ROS, CRT, HMGB1, and NO in collected 4T1 tumors in different groups (G1–G6). c) Operation schematic on in vivo anti‐tumor evaluations. d) Time‐dependent growth profiles of 4T1 tumors harvested from mice that experienced corresponding treatment in different groups (G1–G6), where statistical significance was determined by *t*‐test. e,f) Digital photos (e) and weights (f) of 4T1 tumors harvested from mice that experienced corresponding treatment in different groups (G1–G6) at the end of the experimental period (16 days). g) Time‐dependent survival rates of 4T1 tumor‐bearing mice that experienced corresponding treatment in different groups (G1–G6). h) Heatmap for determining the total differential genes of tumors among the three groups, that is, PBS, ICG@FRMON(US), and ICG/PDE5i@FRMON(US). i) Volcano map for determining the differential genes of 4T1 tumors between PBS and ICG/PDE5i@FRMON(US), j) KEGG analysis for uncovering the affected pathways by above differential genes; and k) GO analysis for finding the affected biological processes (BP), cell components (CC) and molecular functions (MF) by above differential genes. l) Optical microscopic images and LCSM images of 4T1 tumors harvested from mice that experienced corresponding treatment in different groups (G1–G6) at the end of the experimental period (16 days), where tumor slices were stained with HE and *γ*‐H_2_AX immunohistochemistry and TUNEL (green) and Ki‐67 (red) immunofluorescence before observation. Data are expressed as mean ± SD (*n* = 6), and statistical significance was determined by *t*‐test, and *P<0.05, **P<0.01, and ***P<0.001. G1–G6 represent control (PBS), FRMON(US); ICG@FRMON, ICG@FRMON(US), ICG/PDE5i@FRMON and ICG/PDE5i@FRMON(US), respectively; and Scale bar: 50 µm.

By virtue of endosomal escape, more ICG/PDE5i@FRMON nanoparticles enter and remain in the tumor. The accumulation level reaches its peak at 24 h, as evidenced by in vivo and ex vivo fluorescence imaging (Figure S6, Supporting Information); this benefits in vivo anti‐tumor actions. During in vivo ICD test (**Figure** **3**a) on 4T1 tumor‐bearing mice, abundant ROS production is also observed in G4 and G6‐treated groups, suggesting that the intraparticle‐double‐scattering in such sonoimmunity‐engineered ICG/PDE5i@FRMON indeed elevates the US utilization for ROS birth (Figure 3b). Accordingly, the intraparticle‐double‐scattering‐decoded sonogenetics induce the most translocation of CRT and HMGB1 to the outer membrane in 4T1 tumors (Figure 3b). Subsequently, high CRT and HMGB1 expressions favor in vivo DC maturation and impel activation and expansion of effector T (CD8+) including cytotoxic T lymphocytes (CTLs, IFN*γ*+CD8+) in tumor‐draining lymph nodes (TDLNs) (Figures S7a–f and S8, Supporting Information), accompanied with cytokines secreted by matured DCs (IL‐6 and TNF‐*α*) and activated CD8+ T cells (IL‐12 and INF‐*γ*) in the serum, which are up‐regulated (Figure S7g–j, Supporting Information). Akin to the in vitro results, the high expressions of IL‐6 and TNF‐*α* in the serum will also favor CAR‐T expansion in CAR‐T immunotherapy combined therapy. Inspiringly, more NO release from PDE5i‐contained nanoplatforms lays a solid foundation for in vivo vascular normalization and enables the T or CAR‐T cells infiltration in solid tumor (Figure 3b), akin to the in vitro NO test.

furthermore, in vivo anti‐tumor evaluations on 4T1 tumor‐bearing mice were carried out (Figure 3c). Contributed by the direct killing effect, activated systematic immune responses, enhanced ICD, mitigated ITM, promoted CTLs expansion by ROS, and propelled vascular normalization and IB opening by NO. ICG/PDE5i@FRMON(US) (G6) treatment leads to the highest inhibitory rate (Figure 3d and Figure S9, Supporting Information), where the lowest tumor volume and weight and the highest survival rate emerge in G6 (Figure 3e–g). Notably, there was no evident temperature rise and body weight mutation of treated mice during treatment (Figure S10, Supporting Information), as well as no evident tissue injuries to normal tissues (Figure S11, Supporting Information), all of which suggest biosafety. To comprehensively understand this result, RNA sequencing was implemented and G4 (ICG@FRMON(US)) or G6 (ICG/PDE5i@FRMOM(US)) induced significantly‐differential genes were compared to G1 (Control) (Figure 3h). After screening top differential genes between PBS and ICG/PDE5i@FRMON(US) (Figure 3i), some pathways associated with inflammation, immunity, apoptosis, metabolism, homeostasis, and blood vessels were identified due to the emergence of their related differentially‐expressed genes (Figure 3j and Figure S12, Supporting Information), wherein the manipulated biochemical processes are highlighted (Figure 3k). All data suggest that the anti‐tumor mechanism using sonoimmunity‐engineered nanoplatforms influences ROS killing, anti‐tumor immune potentiation, and vascular homeostasis modulation. The subsequent pathological examinations also verify the results. Furthermore, ICG/PDE5i@FRMOM(US) could trigger intraparticle‐double‐scattering‐decoded sonogenetics to destroy DNA, inhibit cell proliferation, and induce cell apoptosis via direct killing and potentiated immunotherapy (Figure 3l); these results re‐observed by comparing the expressions of *γ*‐H_2_AX, Ki‐67, TUNEL, and HE between G1 and G6.

### 2.5 Deep Anti‐Tumor Mechanism Survey of Multiple Actions Enabled by Intraparticle Double‐Scattering‐Decoded Sonogenetics

**Figure 4:**
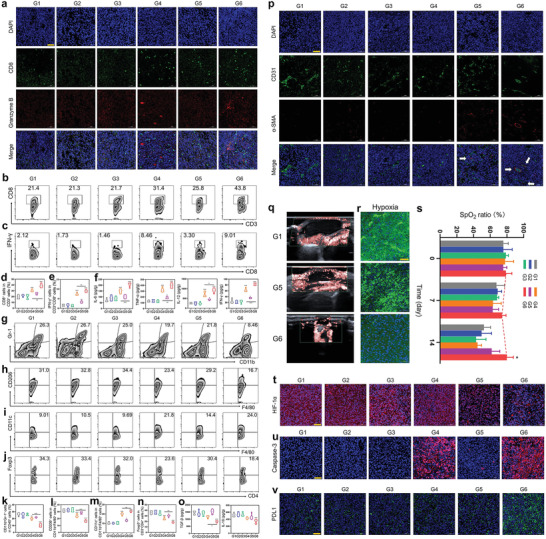
In evaluations on systematic immune response activation, ITM remodeling, and vascular normalization using such sonoimmunity‐engineered nanoplatforms (ICG/PDE5i@FRMON) to elicit intraparticle‐double‐scattering and unlock sonogenetics. a) LCSM images of tumor slices co‐stained with DAPI, CD8, and Granzyme immunofluorescence after 16 days post‐corresponding treatment in different groups (G1–G6). b–e) FCM patterns (b,c) and quantitative percentages (d,e) of CD8+ T cells gating from CD3+ cells (b,d) and cytotoxic T lymphocytes (CTLs, CD8+IFN*γ*+) gating from CD8+CD3+ cells (c,e) in 4T1 tumors after 16 days post‐corresponding treatment in different groups (G1–G6). f) Secretion levels of cytokines including IL‐6, TNF‐*α*, IL‐12, and IFN‐*γ* in 4T1 tumors after 16 days post‐corresponding treatment in different groups (G1–G6). Data are expressed as mean ± SD (*n* = 5). g–n) FCM patterns (g–j) and quantitative percentages (k–n) of Gr‐1+CD11b+CD45+ (MDSCs) (g,k), CD206+CD11b+F4/80+ (M2‐type macrophages) (h,l), CD11c+CD11b+F4/80+ (M1‐type macrophages) (i,m), and FOXP3+CD4+CD3+ (Tregs) (j,n) in 4T1 tumors after 16 days post‐corresponding treatment in different groups (G1–G6). o) Secretion levels of cytokines including TGF‐*β* and IL‐10 in 4T1 tumors after 16 days post‐corresponding treatment in different groups (G1–G6). Data are expressed as mean ± SD (*n* = 5). Statistical significance was determined by ANOVA, and *P<0.05, **P<0.01, and ***P<0.001. p) LCSM images of 4T1 tumor slices co‐stained with DAPI, CD31, and *α*‐SMA immunofluorescence after 16 days post‐corresponding treatment in different groups (G1–G6). q) Color Doppler flow imaging of 4T1 tumors after 14 days post‐corresponding treatment in G1, G3, and G6. r) LCSM images of 4T1 tumor slices after 14 days post‐corresponding treatment in G1, G3, and G6, where green represents hypoxia. s) Oxygen partial pressure of 4T1 tumors after 0, 7, and 14 days post‐corresponding treatment in different groups (G1–G6). t–v) LCSM images of 4T1 tumor slices harvested from 4T1 tumor‐bearing mice after 16 days post‐corresponding in different groups (G1–G6), and before observation, HIF‐1*α* (t), caspase‐3 (u), and PD‐L1 (v) immunofluorescence stainings were implemented. Data are expressed as mean ± SD (*n* = 3). Statistical significance was determined by *t*‐test, and *P<0.05. G1–G6 represent control (PBS), FRMON(US), ICG@FRMON, ICG@FRMON(US), ICG/PDE5i@FRMON, and ICG/PDE5i@FRMON(US), respectively; and Scale bar: 50 µm.

To analyze the rationales of the above multiple actions, various tests of treated tumor tissues were implemented to give direction on addressing the concerns of ICD, ICB, and CAR‐T immunotherapy. The trafficking levels of CD8 and CTLs represented by granzyme B in tumor slices were traced. Akin to results in the above TDLNs, G6 treatment was found to favor the most CD8+ T cells and CTLs to enter the tumor and execute the anti‐tumor actions (**Figure** **4**a). Flow cytometry analysis also showed identical results, where G6 received the highest infiltrated percentages of CD8+ T cells (43.8%) and CTLs (9.01%) (Figure 4b–e). Moreover, G6 treatment also induced the most secretions of cytokines in the tumor (Figure 4f), wherein the high expressions of IL‐6 and TNF‐*α* in the tumor benefited CAR‐T immunotherapy against solid tumors through expanding CTLs and CAR‐T cells. These results revealed that activated immune responses contribute to the repressed tumor growth.

Coincident with systematic immune activation for ICD, ROS is designed to completely reverse ITM including immunosuppressive cells and cytokines. Immune‐resistant cells that render solid tumor immunosuppression were first investigated. Results show that myeloid‐derived suppressor cell (MDSCs, Gr‐1+CD11b+CD45+), M2‐type macrophages (CD206+CD11b+F4/80+) and regulatory T cells (Tregs, FOXP3+CD4+CD3+ or FOXP3+CD4+CD25+) were significantly decreased in G6 (Figure 4g–n, Figures S13 and S14, Supporting Information). Accordingly, immune‐activated M1‐type macrophages (CD11c+CD11b+F4/80+ or CD86+F4/80+CD11b+) were up‐regulated (Figure 4i,m and Figures S14–S16, Supporting Information), and a drop in secretion of immunosuppressive cytokines in G6 was observed (which promotes tumor growth, i.e., TGF‐*β* and IL‐10)^[^ ^44^ ^]^ (Figure 4o). These compelling results uncover the successful and comprehensive ITM mitigation including immunosuppressive cells and cytokines by intraparticle‐double‐scattering‐decoded sonogenetics, which will favorably unlock the ITM‐based imprisonment toward effector T or CAR‐T cells, addressing the non‐persistence of ICB or CAR‐T immunotherapy. Notably, G6 outperforms G4 in mitigating ITM, which could be attributed to the presence of vascular normalization in G6 switching immune desert into the oasis via opening IB and promoting more expanded Car‐T or CTLs infiltrations into the tumor.

To figure out whether in vivo PDE5i‐mediated NO release re‐shaped vascular homeostasis for accelerating vascular normalization, systematic inspections of vascular markers were conducted. Consistent with PCR results (Figure 2i), the overlapping of CD31 with a‐SMA showed that perithelial cells grow and wrap blood vessels (Figure 4p), suggesting successful vascular normalization. Moreover, rich blood perfusion also indicated vascular normalization and IB opening (Figure 4q). In light of the fact that hypoxia alleviation is another characteristic of vascular normalization,^[^ ^32^ ^]^ hypoxia mitigation in different groups was monitored. ICG/PDE5i@FRMON(US) (G6) treatment released NO to promote vascular normalization for eliciting significantly‐decreased hypoxia, while there was no PDE5i‐mediated NO release in G4 to cause evident hypoxia mitigation (Figure 4r). Moreover, the variations of oxygen partial pressure (SpO_2_) were tracked, and abundant oxygen was absorbed in tumors to reverse hypoxia. Importantly, with time, the SpO_2_ ratio gradually escalated, and in contrast, the SpO_2_ ratios in other groups gradually descended (Figure 4s). As well, the down‐regulation of HIF1*α* in G6 elicited hypoxia reversion (Figure 4t), and concurrently induced the high expressions of apoptosis‐, pyroptosis‐, and CTLs‐associated protein (Caspase‐3) (Figure 4u). All these results denote that sonoimmunity‐engineered nanoplatforms successfully remodeled vascular homeostasis and promoted vascular normalization and IB opening, which takes the responsibility for boosting CTLs or CAR‐T infiltration into the tumor to execute immunotherapy.

### 2.6 Potentiated ICB Immunotherapy by Intraparticle‐Double‐Scattering‐Decoded Sonogenetics

**Figure 5:**
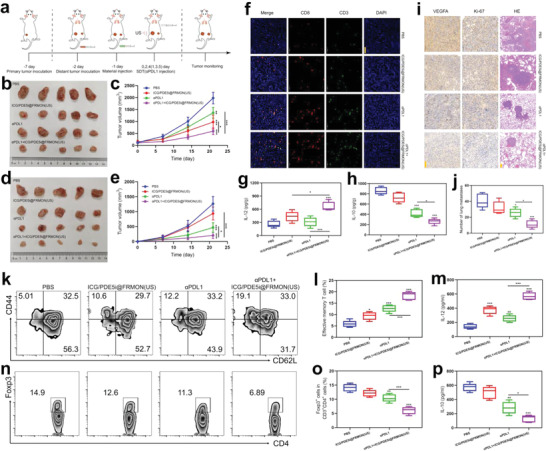
In vivo exploration of ITM mitigation, vascular normalization, and activated systematic immune responses (CTLs replication) in such sonoimmunity‐engineered nanoplatforms (ICG/PDE5i@FRMON) for enhancing ICB therapy. a) Operation schematic on in vivo inhibitory evaluations against primary, distant, and metastasized tumors, where distant tumor on another lateral of lower limp and metastasized tumor on lung via intravenously injecting 4T1 tumor cells were simultaneously established and used. b–e) Digital photos (b,d) and tumor growth profiles (c,e) of primary (b,c) and distant (d,e) tumors in bilaterally‐implanted 4T1 tumor and lung metastasis‐bearing mice that experienced corresponding treatment in different groups, where digital photos were captured at the end of the experimental period (21 days). f) LCSM images of distant 4T1 tumor slices stained with CD8 and CD3 immunofluorescence that were harvested from the above‐mentioned distant and lung metastasis model after 21 days post‐corresponding treatment in different groups. g,h) Secretion levels of IL‐12 (g) and IL‐10 (h) cytokines in distant tumors that were harvested from the above‐mentioned distant and lung metastasis model after 21 days post‐corresponding treatment in different groups. Data are expressed as mean ± SD (*n* = 5). Statistical significance was determined by ANOVA, and *P<0.05, **P<0.01, and ***P<0.001. i) Optical microscopic images of lung metastasis nodules stained with HE, Ki‐67, and VEGFA immunohistochemical antibodies that were harvested from the above‐mentioned distant and lung metastasis model after 21 days post‐corresponding treatment in different groups; and scale bar: 50 µm for VEGFA and Ki‐67 and 200 µm for HE, respectively. j) Quantitative data of lung metastasis nodules on the above‐mentioned distant and lung metastasis model after 21 days post‐corresponding treatment in different groups. k–p) FCM patterns (k,n) and quantitative percentages (l,o) of CD44+CD62L‐ gating from CD8+CD3+ (effective memory T cells, Tem) (k,l) and FOXP3+CD4+ (Tregs) (n,o) in spleen; and (m,p) Secretion levels of cytokines including IL‐12 (m) and IL‐10 (p) in serum, where spleen and serum were harvested from above‐mentioned distant and lung metastasis model after 21 days post‐corresponding treatment in different groups. Data are expressed as mean ± SD (*n* = 5). Statistical significance was determined by ANOVA, and *P<0.05, **P<0.01, and ***P<0.001.

It is worth noting that the intraparticle‐double‐scattering in sonoimmunity‐engineered nanoplatforms decode sonogenetics to up‐regulate the expression of PD‐L1 (Figure 4v), which will benefit anti‐PD‐L1/PD‐1‐based ICB since high PD‐L1 expression is the key factor of ICB. More significantly, the mitigated ITM; activated, systematic, immune, and potentiated ICD; vascular normalization (or IB opening); and CTL expansion are also anticipated to cooperatively contribute to ICB immunotherapy against tumor metastasis. To confirm this, bilaterally‐implanted 4T1 tumors and lung metastasis‐bearing mice were used (**Figure** **5**a). It was found that the marriage of ICG/PDE5i@FRMON(US) with *α*PDL1 outperforms *α*PDL1 alone or ICG/PDE5i@FRMON(US) alone in repressing primary and distant tumors and delaying their growth with the highest inhibitory rate (Figure 5b–e), during which no body weight and temperature variations reflect the treatment biosafety (Figure S17, Supporting Information). Immune‐related analysis revealed that effector T cells (CD8+CD3+) recruitment (Figure 5f), immunostimulation cytokine rise (IL‐12, Figure 5g), and immunosuppressive cytokine decrease (IL‐10, Figure 5h) in the distant tumors are responsible for the distant tumor recession.

Besides repressing distant tumors, *α*PDL1+ICG/PDE5i@FRMON(US) treatment also exerts the most robust inhibitory actions on lung metastasis (Figure 5i,j). In detail, lung nodules were tremendously decreased and tumor cell proliferation and metastasis that are marked with Ki‐67 and VEGFA, respectively, were blockaded (Figure 5i). Deep experiments explain the anti‐metastasis consequences. Naive T cells were found to be first converted into central memory T cells (Tcm, CD44+CD62L+ gating from CD8+CD3+) and eventually into massive effective memory T cells (Tem, CD44+CD62L‐ gating from CD8+CD3+), where naive T cells descend and Tem cells were increased especially when multiple *α*PDL1 injections induced more antigen exposures in distant and lung metastasized tumors (Figure 5k,l, Figures S18 and S19, Supporting Information). These re‐activated memory T cells in the spleen united with immunostimulation IL‐12 cytokine (Figure 5m) to resist tumor metastasis in *α*PDL1+ICG/PDE5i@FRMON(US). Akin to the above results, *α*PDL1+ICG/PDE5i@FRMON(US) treatment elicits the largest magnitude of immunosuppressive Tregs (Figure 5n,o and Figure S19, Supporting Information) and the IL‐10 (Figure 5p) decline in spleen and serum, which also renders lung metastasis unfavorable.

### 2.7 Multiple Actions for Potentiating CAR‐T Immunotherapy against Solid Tumor

**Figure 6:**
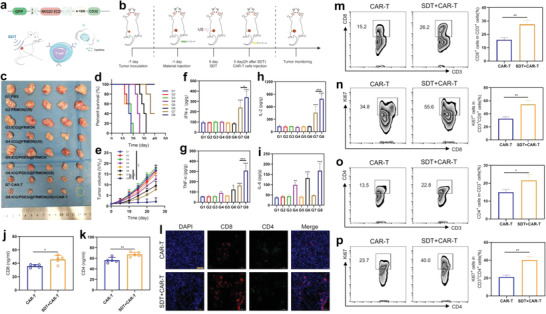
In vivo CAR‐T immunotherapy against solid MDA‐MB‐231 tumor implanted on NSG mice using such intraparticle‐double‐scattering‐encoded sonogenetics via mitigating ITM, promoting vascular normalization and CAR‐T cell replication. a) Design chart and action principle of genetically‐engineered CAR‐T cells, where NKG2D gene was inserted to express the corresponding CAR of NKG2D for binding with MDA‐MB‐231 cancer cells. b) Operation schematic on in vivo CAR‐T immunotherapy against solid MDA‐MB‐231 tumor. c,d) Digital photos (c) and tumor growth profiles (d) of MDA‐MB‐231 tumors in MDA‐MB‐231 tumor‐bearing NSG mice that experienced corresponding treatment in different groups (G1–G8). e) Time‐dependent survival rates of MDA‐MB‐231 tumor‐bearing NSG mice that experienced corresponding treatment in different groups. f–i) Expression levels of cytokines including IFN‐*γ* (f), TNF‐*α* (g), IL‐2 (h), and IL‐6 (i) in serum harvested from MDA‐MB‐231 tumor‐bearing NSG mice. j,k) Expression levels of CD4+ CAR‐T cells (j) and CD8+ CAR‐T cells (k) by ELISA test in serum harvested from MDA‐MB‐231 tumor‐bearing NSG mice. l) LCSM images of MDA‐MB‐231 tumor slices stained with CD4 and CD8 immunofluorescence antibodies harvested from MDA‐MB‐231 tumor‐bearing NSG mice in both groups, that is, CAR‐T and CAR‐T+SDT, and Scale bar: 50 µm. m–p) FCM patterns (right) and quantitative percentages (left) of CD8+CD3+ (m), Ki67+CD8+ (n), CD4+CD3+ (o), and Ki67+CD4+ T cells (p) gating on CD3+ in MDA‐MB‐231 tumors after corresponding treatment in CAR‐T alone (G7) and SDT+CAR‐T (G8) groups, respectively. Data are expressed as mean ± SD (*n* = 3 or 5). Statistical significance was determined by *t*‐test or ANOVA, and *P<0.05, **P<0.01, and ***P<0.001. Note, G1–G8 represents PBS, FRMON (US), ICG@FRMON, ICG@FRMON (US), ICG/PDE5i@FRMON, ICG/PDE5i@FRMON(US), CAR‐T, and ICG/PDE5i@FRMON(US)+CAR‐T+SDT, respectively, where ICG/PDE5i@FRMON(US) is also called as SDT.

The considerable success in augmenting ICB can also reward ICG/PDE5i@FRMON and in turn exemplify the multiple actions and their underlying principles induced by the intraparticle‐double‐scattering‐decoded sonogenetics. On this account, it is confirmed that the compelling multiple actions, for example, systematic immunity activation, ITM mitigation, CTLs expansion, and vascular normalization, can address the three hurdles of CAR‐T immunotherapy for improving the trafficking and persistence of CAR‐T immunotherapy against solid tumors. To validate them, the NKG2D gene was inserted into T cells to express the corresponding CAR of NKG2D allowing NKG2D‐engineered CAR‐T cells to bind with MDA‐MB‐231 cancer cells and induce lysis (**Figure** **6**a). Related experimental procedures are provided in Figure 6b, where immunodeficient NSG mice without matured T, B, and NK cells were used.^[^ ^45^, ^46^ ^]^ SDT that represents ICG/PDE5i@FRMON(US) indeed assists CAR‐T immunotherapy to perform the best in repressing MDA‐MB‐231 tumor associated with delayed tumor growth and shrunk tumor volume, as evidenced by the comparison between G8 (SDT+CAR‐T) and other groups (Figure 6c,d). Simultaneously, the survival rate is significantly prolonged in SDT+CAR‐T group (G8) (Figure 6e) without interfering with body weight and body temperature (Figure S20, Supporting Information). The pathological survey showed that the SDT‐assisted CAR‐T immunotherapy induces a large number of cell deaths (Figure S21, Supporting Information), answering why SDT+CAR‐T group (G8) receives the best anti‐tumor effects.

To further unravel the mechanism, immune‐related indexes were measured, and ICG/PDE5i@FRMON(US)‐enabled SDT failed to promote PD‐L1 expression on MDA‐MB‐231 cells (Figure S22, Supporting Information). Typically, cytokines mattering immune activation and CAR‐T replication (IL‐2, IL‐6, TNF‐*α*, and IFN‐*γ*) are drastically increased, hinting at the replication and function maintenance of CAR‐T cells for executing anti‐tumor actions (Figure 6f–i). Results agree with the expectation that SDT‐activated immune responses indeed facilitate CAR‐T cell propagation since the higher intravascular expressions of CD8 and CD4 in SDT+CAR‐T group than in CAR‐T alone were acquired (Figure 6j–l and Figure S22, Supporting Information). More significantly, IB opening induced by NO‐arised vascular normalization (Figure S23, Supporting Information) and permeability enhancement during SDT allowed more CAR‐T cells (including CD4 and CD8 T cells) to enter and retain in tumors in SDT+CAR‐T group than in CAR‐T alone (Figure 6l,m,o and Figure S24, Supporting Information). Besides enhancing infiltration of CAR‐T cells, SDT+CAR‐T (G8) also considerably elevated the accumulations of Ki67+CD8+ and Ki67+CD4+ gating on CD3+ in comparison to G7 (CAR‐T alone) (Figure 6n,p and Figure S24, Supporting Information), revealing that SDT+CAR‐T promotes the proliferation and expansion of CAR‐T cells. All these results attest that the intraparticle‐double‐scattering‐decoded sonogenetics in sonoimmunity‐engineered SDT indeed can remodel ITM, open IB, and promote CAR‐T expansion for enhancing the trafficking and persistence of Car‐T immunotherapy against solid tumors.

Eventually, we moved to explore the biosafety of such nanoplatforms since excellent biosafety is the premise of clinical translation. Systematic experiments suggest no evident cell death, no blood and biochemical index variation, no body temperature and weight variations even at high doses, and no evident injuries to normal organs (Figure S25, Supporting Information), thus demonstrating the excellent biocompatibility of such sonoimmunity‐engineered nanoplatforms.

## 3 Discussion

As the extensively‐accepted three hurdles of immunotherapy, robust ITM, poor infiltration, and inadequate effector T cells are also the primary limiting factors for CAR‐T immunotherapy (especially for solid tumors). Consequently, we harnessed FRMON which implemented intraparticle‐double‐scattering to elevate the acoustic utilization and further decode sonogenetics to potentiate ROS‐activated sonoimmunity in SDT. The underlying design rationales or strategies provided a distinctive insight into CAR‐T immunotherapy. ROS has been validated to correlate with ICD, systematic immune activation, and complete ITM reversion.^[^ ^29^ ^]^ In this report, the rattle‐type‐structured carriers elevated acoustic utilization via double scattering and unlocked sonogentics to give birth to massive ROS, induce robust ICD, and mitigate ITM (Scheme 1a,d). As one middle part of innate immune, matured DCs were routinely proceeded to present antigens and secrete cytokine promoters to activate and expand T cells.^[^ ^47^ ^]^ Herein, SDT and ICD exposed a lot of antigens exposed to activate DCs maturation for releasing cytokines, and successfully propelled T cell replication (Scheme 1a,d).

NO as a vascular relaxation agent plays an important role in vascular homeostasis modulation for enhancing T cell infiltration.^[^ ^32^, ^34^ ^]^ Enlightened by it, the entrapped PDE5i was designed to release NO via the iNOs/cGMP pathway and then harnessed to switch disorder blood vessels into normalized ones, conferring more effector CD8+ T or CAR‐T cells’ with open IB to enter tumors (Scheme 1b,d). Collectively, IB opening, CTL replication, and ITM remodeling removed the difficulties that current immunotherapy was subjected to, thus addressing the non‐persistence of ICB or CAR‐T immunotherapy. Furthermore, ICB immunotherapy also indirectly verifies the successful CTL replication, ITM remodeling, and IB opening, ensuring enhanced CAR‐T immunotherapy (Scheme 1c,d).

In summary, we engineered a sonoimmunity‐inspired SDT agent that can unlock intraparticle‐double‐scattering‐decoded sonogenetics for resolving the encountered three hurdles of CAR‐T immunotherapy. A series of experiments revealed that SDT could evidently elevate ultrasound utilization via double backscattering for producing abundant ROS. The significantly‐accumulated ROS directly kill tumors, induces robust ICD, activates systematic immune responses, mitigates ITM including immunosuppressive cells or cytokines, and releases NO for vascular normalization and IB opening; all of these effects have been validated to remove the ITM‐induced imprisonment toward CAR‐T cells, promote CAR‐T expansion, and facilitate their entry into the solid tumor for exerting the persistent anti‐tumor actions against solid tumors. Moreover, this general method was successfully harnessed to potentiate ICB featuring anti‐metastasis, thus holding high clinical translation potential.

## 4 Experimental Section

Materials and experimental details are included in Supporting Information.

## 5 Ethic Statement

All animal experiments were approved by the Laboratory Animal Center in Affiliated Cancer Hospital of Guangxi Medical University with an approval number (LW2021092).

## Conflict of Interest

The authors declare no conflict of interest.

## Author Contributions

D.W. and M.Z. contributed equally to this work. K.Z. designed the project. K.Z. conceived and proposed the novelty and paper structure. D.W., M.Z., Y.Z., G.Q., J.C., X.Z., C.K., X.L., X.L., L.D., and C.F. performed the experiments, and D.W. analyzed the data. K.Z. and D.W. wrote and revised the manuscript. J.L. and K.Z. supported the project, and J.L., T.L., and K.Z. supervised the project and all authors commented on this manuscript.

## Supporting information

Supporting Information

## Data Availability

The data that support the findings of this study are available from the corresponding author upon reasonable request.

## References

[1] G. Giordano‐Attianese , P. Gainza , E. Gray‐Gaillard , E. Cribioli , S. Shui , S. Kim , M.‐J. Kwak , S. Vollers , A. D. J. C. Osorio , P. Reichenbach , J. Bonet , B.‐H. Oh , M. Irving , G. Coukos , B. E. Correia , Nat. Biotechnol. 2020, 38, 426. 32015549 10.1038/s41587-019-0403-9

[2] K. Watanabe , S. Kuramitsu , A. D. Posey Jr. , C. H. June , Front. Immunol. 2018, 9, 2486. 30416506 10.3389/fimmu.2018.02486 PMC6212550

[3] Y. J. Xie , M. Dougan , N. Jailkhani , J. Ingram , T. Fang , L. Kummer , N. Momin , N. Pishesha , S. Rickelt , R. O. Hynes , H. Ploegh , Proc. Natl. Acad. Sci. U. S. A. 2019, 116, 7624. 30936321 10.1073/pnas.1817147116 PMC6475367

[4] I. Scarfo , M. V. Maus , J. Immunother. Cancer 2017, 5, 28. 28331617 10.1186/s40425-017-0230-9 PMC5359946

[5] L. Labanieh , R. G. Majzner , C. L. Mackall , Nat. Biomed. Eng. 2018, 2, 377. 31011197 10.1038/s41551-018-0235-9

[6] M. Martinez , E. K. Moon , Front. Immunol. 2019, 10, 128. 30804938 10.3389/fimmu.2019.00128 PMC6370640

[7] P. Wolf , J. Alzubi , C. Gratzke , T. Cathomen , Nat. Rev. Urol. 2021, 18, 556. 34239139 10.1038/s41585-021-00488-8

[8] M. Klichinsky , M. Ruella , O. Shestova , X. M. Lu , A. Best , M. Zeeman , M. Schmierer , K. Gabrusiewicz , N. R. Anderson , N. E. Petty , K. D. Cummins , F. Shen , X. Shan , K. Veliz , K. Blouch , Y. Yashiro‐Ohtani , S. S. Kenderian , M. Y. Kim , R. S. O'Connor , S. R. Wallace , M. S. Kozlowski , D. M. Marchione , M. Shestov , B. A. Garcia , C. H. June , S. Gill , Nat. Biotechnol. 2020, 38, 947. 32361713 10.1038/s41587-020-0462-y PMC7883632

[9] L. Ma , T. Dichwalkar , J. Y. H. Chang , B. Cossette , D. Garafola , A. Q. Zhang , M. Fichter , C. Wang , S. Liang , M. Silva , S. Kumari , N. K. Mehta , W. Abraham , N. Thai , N. Li , K. D. Wittrup , D. J. Irvine , Science 2019, 365, 162. 31296767 10.1126/science.aav8692 PMC6800571

[10] M. Chmielewski , H. Abken , Cell Rep. 2017, 21, 3205. 29241547 10.1016/j.celrep.2017.11.063

[11] L. Giuffrida , K. Sek , M. A. Henderson , J. Lai , A. X. Y. Chen , D. Meyran , K. L. Todd , E. V. Petley , S. Mardiana , C. Molck , G. D. Stewart , B. J. Solomon , I. A. Parish , P. J. Neeson , S. J. Harrison , L. M. Kats , I. G. House , P. K. Darcy , P. A. Beavis , Nat. Commun. 2021, 12, 3236. 34050151 10.1038/s41467-021-23331-5 PMC8163771

[12] S. Li , N. Siriwon , X. Zhang , S. Yang , T. Jin , F. He , Y. J. Kim , J. Mac , Z. Lu , S. Wang , X. Han , P. Wang , Clin. Cancer Res. 2017, 23, 6982. 28912137 10.1158/1078-0432.CCR-17-0867

[13] A. Rodriguez‐Garcia , R. C. Lynn , M. Poussin , M. A. Eiva , L. C. Shaw , R. S. O'Connor , N. G. Minutolo , V. Casado‐Medrano , G. Lopez , T. Matsuyama , D. J. Powell Jr. , Nat. Commun. 2021, 12, 877. 33563975 10.1038/s41467-021-20893-2 PMC7873057

[14] L. Jin , H. Tao , A. Karachi , Y. Long , A. Y. Hou , M. Na , K. A. Dyson , A. J. Grippin , L. P. Deleyrolle , W. Zhang , D. A. Rajon , Q. J. Wang , J. C. Yang , J. L. Kresak , E. J. Sayour , M. Rahman , F. J. Bova , Z. Lin , D. A. Mitchell , J. Huang , Nat. Commun. 2019, 10, 4016. 31488817 10.1038/s41467-019-11869-4 PMC6728370

[15] K. Reinhard , B. Rengstl , P. Oehm , K. Michel , A. Billmeier , N. Hayduk , O. Klein , K. Kuna , Y. Ouchan , S. Woll , E. Christ , D. Weber , M. Suchan , T. Bukur , M. Birtel , V. Jahndel , K. Mroz , K. Hobohm , L. Kranz , M. Diken , K. Kuhlcke , O. Tureci , U. Sahin , Science 2020, 367, 446. 31896660 10.1126/science.aay5967

[16] A. J. Hou , L. C. Chen , Y. Y. Chen , Nat. Rev. Drug Discovery 2021, 20, 531. 33972771 10.1038/s41573-021-00189-2

[17] J. Chen , I. F. Lopez‐Moyado , H. Seo , C.‐W. J. Lio , L. J. Hempleman , T. Sekiya , A. Yoshimura , J. P. Scott‐Browne , A. Rao , Nature 2019, 567, 530. 30814732 10.1038/s41586-019-0985-x PMC6546093

[18] R. Grosser , L. Cherkassky , N. Chintala , P. S. Adusumilli , Cancer Cell 2019, 36, 471. 31715131 10.1016/j.ccell.2019.09.006 PMC7171534

[19] G. Agliardi , A. R. Liuzzi , A. Hotblack , D. De Feo , N. Nunez , C. L. Stowe , E. Friebel , F. Nannini , L. Rindlisbacher , T. A. Roberts , R. Ramasawmy , I. P. Williams , B. M. Siow , M. F. Lythgoe , T. L. Kalber , S. A. Quezada , M. A. Pule , S. Tugues , K. Straathof , B. Becher , Nat. Commun. 2021, 12, 444. 33469002 10.1038/s41467-020-20599-x PMC7815781

[20] K. Zhang , Y. Fang , Y. He , H. Yin , X. Guan , Y. Pu , B. Zhou , W. Yue , W. Ren , D. Du , H. Li , C. Liu , L. Sun , Y. Chen , H. Xu , Nat. Commun. 2019, 10, 5380. 31772164 10.1038/s41467-019-13115-3 PMC6879564

[21] H. Mei , X. Zhang , S. Cai , X. Zhang , Y. Zhang , Z. Guo , W. Shi , R. Chu , K. Zhang , J. Cao , B. He , Nano Today 2021, 41, 101305.

[22] R. Fu , H. Li , R. Li , K. McGrath , G. Dotti , Z. Gu , Adv. Funct. Mater. 2021, 31, 2009489.

[23] E. A. Ogunnaike , A. Valdivia , M. Yazdimamaghani , E. Leon , S. Nandi , H. Hudson , H. Du , S. Khagi , Z. Gu , B. Savoldo , F. S. Ligler , S. Hingtgen , G. Dotti , Sci. Adv. 2021, 7, eabg5841. 34613775 10.1126/sciadv.abg5841 PMC8494441

[24] Y. Wu , Y. Liu , Z. Huang , X. Wang , Z. Jin , J. Li , P. Limsakul , L. Zhu , M. Allen , Y. Pan , R. Bussell , A. Jacobson , T. Liu , S. Chien , Y. Wang , Nat. Biomed. Eng. 2021, 5, 1336. 34385696 10.1038/s41551-021-00779-w PMC9015817

[25] N. T. Nguyen , K. Huang , H. Zeng , J. Jing , R. Wang , S. Fang , J. Chen , X. Liu , Z. Huang , M. J. You , A. Rao , Y. Huang , G. Han , Y. Zhou , Nat. Nanotechnol. 2021, 16, 1424. 34697491 10.1038/s41565-021-00982-5 PMC8678207

[26] P. Yang , F. Zhao , J. Ding , J. Guo , W. Shi , C. Wang , X. Hu , Chem. Mater. 2014, 26, 2121.

[27] K. Zhang , H. Chen , X. Guo , D. Zhang , Y. Zheng , H. Zheng , J. Shi , Sci. Rep. 2015, 5, 8766. 25739832 10.1038/srep08766 PMC4350106

[28] C. Chen , X. Ni , S. Jia , Y. Liang , X. Wu , D. Kong , D. Ding , Adv. Mater. 2019, 31, 1904914. 10.1002/adma.201904914 31696981

[29] Y. Yin , X. Jiang , L. Sun , H. Li , C. Su , Y. Zhang , G. Xu , X. Li , C. Zhao , Y. Chen , H. Xu , K. Zhang , Nano Today 2021, 36, 101009.

[30] H. Wu , H. Li , Y. Liu , J. Liang , Q. Liu , Z. Xu , Z. Chen , X. Zhang , K. Zhang , C. Xu , Bioact Mater 2022, 13, 223. 10.1016/j.bioactmat.2021.10.048 PMC8843980 35224304

[31] T. Luo , D. Wang , L. Liu , Y. Zhang , C. Han , Y. Xie , Y. Liu , J. Liang , G. Qiu , H. Li , D. Su , J. Liu , K. Zhang , Adv. Sci. 2021, 8, 2101065. 10.1002/advs.202101065 PMC8498884 34369112

[32] Y. C. Sung , P. R. Jin , L. A. Chu , F. F. Hsu , M. R. Wang , C. C. Chang , S. J. Chiou , J. T. Qiu , D. Y. Gao , C. C. Lin , Y. S. Chen , Y. C. Hsu , J. Wang , F. N. Wang , P. L. Yu , A. S. Chiang , A. Y. Wu , J. J. Ko , C. P. Lai , T. T. Lu , Y. Chen , Nat. Nanotechnol. 2019, 14, 1160. 31740794 10.1038/s41565-019-0570-3

[33] P. Carmeliet , R. K. Jain , Nat. Rev. Drug Discovery 2011, 10, 417. 21629292 10.1038/nrd3455

[34] S. Kashiwagi , K. Tsukada , L. Xu , J. Miyazaki , S. V. Kozin , J. A. Tyrrell , W. C. Sessa , L. E. Gerweck , R. K. Jain , D. Fukumura , Nat. Med. 2008, 14, 255. 18278052 10.1038/nm1730

[35] K. Zhang , H.‐Y. Li , J.‐Y. Lang , X.‐T. Li , W.‐W. Yue , Y.‐F. Yin , D. Du , Y. Fang , H. Wu , Y.‐X. Zhao , C. Xu , Adv. Funct. Mater. 2019, 29, 1905124.

[36] K. Zhang , H. Chen , P. Li , X. Bo , X. Li , Z. Zeng , H. Xu , ACS Appl. Mater. Interfaces 2015, 7, 18590. 26245739 10.1021/acsami.5b04999

[37] K. Zhang , H. Chen , Y. Zheng , Y. Chen , M. Ma , X. Wang , L. Wang , D. Zeng , J. Shi , J. Mater. Chem. 2012, 22, 12553.

[38] T. Wang , X. Xu , K. Zhang , Curr. Cancer Drug Targets 2021, 21, 545. 33618647 10.2174/1568009621666210219101552

[39] D. Fukumura , S. Kashiwagi , R. K. Jain , Nat. Rev. Cancer 2006, 6, 521. 16794635 10.1038/nrc1910

[40] K. Zhang , H. Xu , X. Jia , Y. Chen , M. Ma , L. Sun , H. Chen , ACS Nano 2016, 10, 10816. 28024356 10.1021/acsnano.6b04921

[41] M. M. Wang , H. M. Liu , L. Li , Y. Y. Cheng , Nat. Commun. 2014, 5, 3053. 24407172 10.1038/ncomms4053

[42] J. Xu , J. Lv , Q. Zhuang , Z. J. Yang , Z. Q. Cao , L. G. Xu , P. Pei , C. Y. Wang , H. F. Wu , Z. L. Dong , Y. Chao , C. Wang , K. Yang , R. Peng , Y. Y. Cheng , Z. Liu , Nat. Nanotechnol. 2020, 15, 1043. 33139933 10.1038/s41565-020-00781-4

[43] R. D. Song , T. L. Li , J. Y. Ye , F. Sun , B. Hou , M. Saeed , J. Gao , Y. J. Wang , Q. W. Zhu , Z. Xu , H. J. Yu , Adv. Mater. 2021, 33, 2101155. 10.1002/adma.202101155 34170581

[44] K. Twumasi‐Boateng , J. L. Pettigrew , Y. Y. E. Kwok , J. C. Bell , B. H. Nelson , Nat. Rev. Cancer 2018, 18, 419. 29695749 10.1038/s41568-018-0009-4

[45] H. Jetani , A. Navarro‐Bailon , M. Maucher , S. Frenz , C. Verbruggen , A. Yeguas , M. B. Vidriales , M. Gonzalez , J. R. Saborido , S. Kraus , K. Mestermann , S. Thomas , H. Bonig , M. Luu , R. Monjezi , D. Mougiakakos , M. Sauer , H. Einsele , M. Hudecek , Blood 2021, 138, 1830. 34289026 10.1182/blood.2020009192 PMC9642786

[46] D. Li , N. Li , Y. F. Zhang , H. Y. Fu , M. Q. Feng , D. Schneider , L. Su , X. L. Wu , J. Zhou , S. Mackay , J. Kramer , Z. J. Duan , H. J. Yang , A. Kolluri , A. M. Hummer , M. B. Torres , H. Zhu , M. D. Hall , X. L. Luo , J. Q. Chen , Q. Wang , D. Abate‐Daga , B. Dropublic , S. M. Hewitt , R. J. Orentas , T. F. Greten , M. Ho , Gastroenterology 2020, 158, 2250. 32060001 10.1053/j.gastro.2020.02.011 PMC7282931

[47] D. Fukurnura , J. Kloepper , Z. Amoozgar , D. G. Duda , R. K. Jain , Nat. Rev. Clin. Oncol. 2018, 15, 325. 29508855 10.1038/nrclinonc.2018.29 PMC5921900

