# Supplementary material for: Intraparticle Double‐Scattering‐Decoded Sonogenetics for Augmenting Immune Checkpoint Blockade and CAR‐T Therapy
Source: Adv Sci (Weinh). 2022 Sep 25;9(32):2203106. doi: 10.1002/advs.202203106 (PMC9661857; doi:10.1002/advs.202203106)
Supplement: Supplementary file 1 — Supporting Information [file ADVS-9-2203106-s001.pdf]

ADVANCED SCIENCE
Open Access

Supporting Information

for *Adv. Sci.*, DOI 10.1002/advs.202203106

Intraparticle Double-Scattering-Decoded Sonogenetics for Augmenting Immune Checkpoint Blockade and CAR-T Therapy

Duo Wang, Mengqi Zhang, Yan Zhang, Guanhua Qiu, Jie Chen, Xiaoqi Zhu, Cunqing Kong, Xiuxin Lu, Xiayi Liang, Lixia Duan, Chao Fang, Junjie Liu, Kun Zhang* and Tao Luo**

Intraparticle Double-Scattering-Decoded Sonogenetics for Augmenting Immune Checkpoint Blockade and CAR-T Therapy

Duo Wang,# Mengqi Zhang,# Yan Zhang, Guanhua Qiu, Jie Chen, Xiaoqi Zhu, Cunqing Kong, Xiuxin Lu, Xiayi Liang, Lixia Duan, Chao Fang, Junjie Liu, Kun Zhang* and Tao Luo**

Dr. D. Wang, Dr. M. Zhang, Dr. G. Qiu, Dr. X. Zhu, Dr. C. Kong, Dr. X. Lu, Dr. T. Luo and Prof. J. Liu

Department of Medical Ultrasound, Department of Interventional Therapy and Department of Gastrointestinal Surgery, Guangxi Medical University Cancer Hospital, Guangxi Medical University. No. 71 Hedi Road, Nanning 530021, P. R. China. * Email: Junjie Liu (liujunjie@gxmu.edu.cn) and Tao Luo (luotao@gxmu.edu.cn)

Dr. Y. Zhang, Prof. J. Chen, Dr. X. Liang, Dr. L. Duan, Dr. C. Fang and Prof. K. Zhang

Central Laboratory and Ultrasound Research and Education Institute, Shanghai Tenth People's Hospital, Tongji University School of Medicine. No. 301 Yan-chang-zhong Road, Shanghai, 200072, P. R. China. * Email: Kun Zhang (zhang1986kun@126.com)

Dr. C. Fang and Prof. K. Zhang

National Center for International Research of Bio-targeting Theranostics, Guangxi Key Laboratory of Bio-targeting Theranostics, Collaborative Innovation Center for Targeting Tumor Diagnosis and Therapy, Guangxi Medical University. No. 22 Shuangyong Road, Nanning 530021, P. R. China.

# The authors contributed equally to this work.

Part A: Experimental section

FRMON and FHMON synthesis

FRMON was obtained *via* a classic in-situ hydrophobic layer protected selective etching method.[1-2] In detail, deionized water (DIW, 5 mL), ammonia solution (1.6 mL, Acros, China) and absolute ethyl alcohol (36 mL, Acros, China) were gently mixed in wild-mouth bottle and then pre-heated in a thermostatic water bath for 30 min at 30 ℃ with a stirring velocity of 300 rpm. After that, 3 mL of tetraethyl orthosilicate (TEOS, Macklin, China) was slowly added to above mixed solution using the pipette, and the hydrolysis reaction proceeded for 45 minutes at 30 ℃ at the stirring velocity of 300 rpm, obtaining solid silica nanospheres (s-SiO_2). Afterwards, another mixture containing 2.5 mL of TEOS and 1 mL of PDES (J&K Scientific, China) was dropwise injected using a microtitrator and coated nanospheres with the hydrophobic hybrid layer for 1 h reaction at 25 °C with the stirring velocity of 450 rpm. Subsequently, the mixed solution was centrifuged at 25 °C for 10 min (velocity: 12000 rpm) and washed three times with DIW (25 mL). After centrifugation for 5 min (velocity: 5000 rpm), the supernatant was discarded, and precipitates were collected. Afterwards, 3.18 g of Na_2CO_3 (Macklin, China) was dissolved in 50 mL of DIW and after complete dissolution, and the collected nanoprecipitates in above step were re-dispersed in the Na_2CO_3 solution *via* ultrasonic vibration. After etching for 7 minutes at 60 °C (250 rpm stirring rate), the final FRMON were harvested *via* centrifugation at 3000 rpm for 5 min, and subsequently 25 mL of DIW was added to re-suspend FRMON for washing FRMON twice *via* centrifugation (5000 rpm) for 5 min.

As for FHMON synthesis, 75 mL of Na_2CO_3 solution (0.6 M) was added to etch the silica inner core, where other conditions were identical to that of FRMON.

ICG@FRMON and ICG/PDE5i@FRMON synthesis

Indocyanine green (ICG, Aladdin, China) and/or phosphodiesterase 5 inhibitor (PDE5i, MCE, USA) solution were pre-prepared in advance with concentrations at 10 mg/mL and 20 mg/mL, respectively. During continuously stirring (velocity: 500 rpm), FRMON sample was added into above mixture using a microtiter within 8 h to obtain the FRMON dispersion (5 mg/mL), and reaction for another 24 h was proceeded at room temperature. After centrifugation (5 min, velocity: 5000 rpm), the precipitates were collected and washed twice with anhydrous ethanol and once with DIW. Finally, the precipitates were re-suspended and stored in PBS solution for further use.

Characterizations

Transmission electron microscope (TEM) images were obtained on -FEI Tecnai F20 (FEI, USA). Dynamic light scattering (DLS) and zeta potential determination were carried on Zetasizer Nano ZS90 (Malvern, UK). N_2 adsorption and desorption isotherms and pore diameter distribution were measured on ASAP 2460 (Micromeritics USA). UV-Vis spectra and FTIR spectra were recorded on Varioskan LUX (ThermoFisher Scientific, China) and Scientific Nicolet iS5 (Thermo, USA), respectively. Element mapping and electron spin resonance (ESR) spectra were obtained on Sigma300 (Zeiss, Germany) and Bruker EMXplus (Bruker, Germany), respectively.

Cell lines and animals

Murine breast cancer cell line (4T1), human breast cancer cell lineage (MDA-MB-231), human umbilical vein endothelial cell (HUVECs) lineage and murine normal liver lineage (AML-12) were purchased from the ATCC (Manassas, VA, USA). The cells were cultured in a humidified incubator at 37 °C and 5% CO_2 atmosphere under the optimum conditions. BALB/C and NSG mice were purchased from SPF Biotechnology Inc. (Beijing, China).

Detection of intracellular ROS production

4T1 cells and MDA-MB-231 cells in the logarithmic growth phase were seeded in six-well plates at a density of 1×10^6 cells per well for 24 h, respectively. Six groups were divided, *i.e.*, G1: control (PBS), G2: FRMON(US); G3: ICG@FRMON, G4: ICG@FRMON(US), G5: ICG/PDE5i@FRMON and G6: ICG/PDE5i@FRMON(US). Afterwards, identical carrier dose (100 μg/mL FRMON, ICG: 4.86 μg/mL, PDE5i: 7.11 μg/mL) was used treat cells in each group for 8 h. Next, DCFH-DA probes (1:1000 dilution folds, S0033M, Beyotime, China) were added cells before SDT treatment (US irradiations parameters were given as follows: power density = 1 W/cm^2, duty cycle = 50%, transducer = 1 MHz, duration = 3 min, agar gel with a thickness of 0.5 cm was placed between the plates and ultrasound probe). Then, the six-well plate was placed in a cell incubator (37 °C, 5% CO_2) and incubated for 10 minutes in the darkness, and washed with PBS three times. The ROS production in the cells was observed and recorded on an inverted fluorescence microscope (Excitation wavelength (λ_{ex}) = 488 nm, emission wavelength (λ_{em}) = 525 nm; and green fluorescence signal represents ROS).

Detection of intracellular NO production

The 4T1 cells and MDA-MB-231 cells in the logarithmic growth phase were seeded in a six-well plate at a density of 1×10^6 cells per well for 24 h, respectively. Six groups were set, *e.g.*, G1: control (PBS), G2: FRMON(US); G3: ICG@FRMON, G4: ICG@FRMON(US); G5: ICG/PDE5i@FRMON and G6: ICG/PDE5i@FRMON(US), wherein the corresponding sample and treatment in G1-G6 were carried out for 8 h (dose: 100 μg/mL FRMON, ICG: 4.86 μg/mL, PDE5i: 7.11 μg/mL). Next, DAF-FM DA (1:1000 dilution, S0019, Beyotime, China) were added before SDT treatment ((US irradiations parameters were given as follows: power density = 1 W /cm^2, duty cycle = 50%, transducer = 1 MHz, duration =1 min, agar gel with a thickness of 0.5 cm was placed between the

plates and ultrasound probe). Afterwards, the six-well plates were placed in a cell incubator (37 °C, 5% CO_2) and incubated for 10 minutes in the darkness, and then washed with PBS three times. The NO production in the cells was observed and recorded on an inverted fluorescence microscope, wherein green florescence signal representing NO was detected at λ_{ex} = 495 nm and λ_{em} = 515 nm.

Identification between dead and live cells after Calcein AM/PI staining

4T1 cells in the logarithmic growth phase were respectively seeded in six-well plates at a density of 1×10^6 cells per well for 24 h. Group setting: G1: control (PBS), G2: FRMON(US); G3: ICG@FRMON, G4: ICG@FRMON(US); G5: ICG/PDE5i@FRMON and G6: ICG/PDE5i@FRMON(US).The survival/necrosis rate of cells were measured after drug treatment (dose: 100 μg/mL FRMON, ICG: 4.86 μg/mL, PDE5i: 7.11 μg/mL) and sonodynamic therapy (US irradiations parameters were unchanged) in each group. Then, the Calcein AM/ propidium iodide (PI) diluted at 1:1000 (C2015M, Beyotime, China) was added to the cells and incubated in a 5% CO_2 incubator for 10 min at 37 ℃ in the darkness and washed with PBS three times. The cytoplasmic green fluorescence representing live cells was recorded at the excitation wavelength of 494 nm and emission wavelength of 517 nm, and red fluorescence representing dead cells was captured at the excitation wavelength of 535 nm and emission wavelength of 617 nm.

Cell viability assay

4T1 cells in the logarithmic growth phase were seeded in a 96-well plate for 24 h (density: 5000 cells/well). Six groups (*i.e.*, G1: control (PBS), G2: FRMON(US); G3: ICG@FRMON, G4: ICG@FRMON(US); G5: ICG/PDE5i@FRMON and G6: ICG/PDE5i@FRMON(US)) were set. Afterwards, corresponding treatments in G1-G6 (dose: 100 μg/mL FRMON, ICG: 4.86 μg/mL, PDE5i: 7.11 μg/mL) for 8 h were implemented and then SDT was performed in each group (US

irradiations parameters were set as follows: power density = 1 W/cm^2, duty cycle = 50%, transducer = 1 MHz, duration =1 min, agar gel with a thickness of 0.5 cm was placed between the plates and ultrasound probe). Cell proliferation was evaluated with a Cell Counting Kit-8 (C0038, Beyotime, China) according to the manufacturer's instructions. Briefly, CCK8 solution (10 μL) was added into each well, incubated at 37 °C for 1 h, and then the absorbance at 450 nm was measured.

Colony formation assay

Identical grouping was made, G1: control (PBS), G2: FRMON(US); G3: ICG@FRMON, G4: ICG@FRMON(US); G5: ICG/PDE5i@FRMON and G6: ICG/PDE5i@FRMON(US). 4T1 cells in the logarithmic growth phase were trypsinized, and the cell concentration was calculated after re-suspending in the complete medium (10% serum, 1% penicillin-streptomycin), then seeded in a 6-well plate at a density of 500 cells/well. Afterwards, corresponding treatments in G1-G6 (dose: 100 μg/mL FRMON, ICG: 4.86 μg/mL, PDE5i: 7.11 μg/mL) for 8 h were implemented and then SDT was performed in each group (US irradiations parameters were set as follows: power density = 1 W/cm^2, duty cycle = 50%, transducer = 1 MHz, duration =1 min, agar gel with a thickness of 0.5 cm was placed between the plates and ultrasound probe). The cell colonies were cultured for 14 days and the media were re-freshed every 3 days. When the colonies were formed, they were washed with PBS twice, and then 1 mL of 4% paraformaldehyde was added to each well to fix cells for 30 min, followed by staining with 1% crystal violet. After drying, photographic recording and data analysis were performed in sequence on the inverted microscope and Image J software, respectively.

Phagocytosis test

4T1 cells (1×10^5 /well) were seeded in a special culture dish, and after overnight incubation, the cell nuclei were stained with Hoechst 33342 (10 μg/mL, C1025, Beyotime, China) for 15 minutes and

used PBS to wash the dishes three times. Then, lysosomal probe (BB3603, Bestbio, China) with a 1:1000 dilution fold was added and incubated in the darkness for 15 minutes, followed by washing again and treatment with ICG/PDE5i@FRMON. Finally, the cell morphology was observed on the laser confocal scanning microscopy (LCSM, Leica SP8, Germany).

Commercial sources of antibodies and ELISA kits for flow cytometry (FCM), Immunohistochemistry (IHC), immunofluorescence (IF) staining and cytokine tests.

Table 1. Antibody information in FCM analysis

Name	Art.No.	Company	Country
Anti-Mouse CD45	103116	Biolegend	USA
Anti-Mouse CD3	100306	Biolegend	USA
Anti-Mouse CD8	100722	Biolegend	USA
Anti-Mouse CD4	100559	Biolegend	USA
Anti-Mouse FOXP3	17-5773-82	Thermo	USA
Anti-Mouse CD25	102008	Biolegend	USA
Anti-Mouse CD62L	104412	Biolegend	USA
Anti-Mouse Gr-1	108445	Biolegend	USA
Anti-Mouse IFN -γ	505830	Biolegend	USA
Anti-Mouse F4/80	123122	Biolegend	USA
Anti-Mouse MHC II	107606	Biolegend	USA
Anti-Mouse CD11c	117310	Biolegend	USA
Anti -Mouse CD11b	101208	Biolegend	USA
Anti-Mouse CD206	141720	Biolegend	USA
Anti-Mouse CD80	104708	Biolegend	USA
Anti-Mouse CD86	105040	Biolegend	USA
Anti-Human CD45	304014	Biolegend	USA
Anti-Human CD3	317336	Biolegend	USA
Anti-Human CD8	344712	Biolegend	USA
Anti-Human CD4	317444	Biolegend	USA
Anti-Human Ki-67	350506	Biolegend	USA

Table 2. ELISA kits information

Name	Art.No.	Company	Country
Mouse IL-6	EMC004	Neobioscience	China
Mouse IL-10	EMC005	Neobioscience	China
Mouse IL-12	EMC109	Neobioscience	China
Mouse TNF-α	EMC102a	Neobioscience	China
Mouse IFN-γ	EMC101g	Neobioscience	China
Mouse TGF-β	EMC107b	Neobioscience	China
Human IL-2	EHC003	Neobioscience	China
Human TNF-α	EHC103a	Neobioscience	China
Human IFN-γ	EHC102g	Neobioscience	China
Human CD8	PH098875	PYRAM	China
Human CD4	PH098883	PYRAM	China

Table 3. Antibody information in IHC and IF staining

Name	Art.No.	Company	Country
Anti-HMGB1	GB11103	Servicebio	China
Anti-CRT	AF1666	Beyotime	China
Anti-Ki-67	GB111499	Servicebio	China
Anti-CD3	GB111337	Servicebio	China
Human Anti-CD8	GB11068-1	Servicebio	China
Mouse Anti-CD8	GB11068	Servicebio	China
Human Anti-CD4	GB11064-1	Servicebio	China
Anti-Caspase-3	GB11532	Servicebio	China
Anti-PDL1	GB11339	Servicebio	China
Anti-HIF-1α	GB13031-1	Servicebio	China
Anti-Granzyme B	Ab255598	Abcam	USA
Anti-VEGFA	AF5131	Affnity	China
Anti-α-SMA	GB13044	Servicebio	China
Anti-CD31	GB12063	Servicebio	China
Anti-CD86	GB13585	Servicebio	China
Anti-F4/80	GB113373	Servicebio	China
DAR-1(NO probe)	Ab145388	Abcam	USA

Quantitative real-time polymerase chain reaction (qRT-PCR)

Three groups were set, *i.e.*, PBS, PDE5i@FRMON, PDE5i@FRMON(US), and HUVECs in the

logarithmic growth stage were inoculated in 6-well plates at 1×10^6 per well, and incubated for 24 h. Afterwards, corresponding treatments in the three groups (dose: 100 μg/mL FRMON, ICG: 4.86 μg/mL, PDE5i: 7.11 μg/mL) for 8 h were implemented and then SDT (i.e., ICG/PDE5i@FRMON(US)) was performed in each group (US irradiations parameters were set as follows: power density = 1 W/cm^2, duty cycle = 50%, transducer = 1 MHz, duration =1 min, agar gel with a thickness of 0.5 cm was placed between the plates and ultrasound probe). After incubating for another 24 h, HUVEC cells in the three groups were digested with trypsin and collected, and then 1 mL of Trizol (Thermo Scientific, USA) was added to lyse the cells to extract RNA according to the manufacturer's procedures. Total RNA (500 ng) was reversely transcribed using Mix-X miRNA First-Strand Synthesis Kit (RR047, Takara, Japan) to obtain cDNA. Quantitative real-time PCR was carried out with SYBR green PCR Master Mix that followed the standard protocols, and related analysis was implemented on the Step One Plus qRT-PCR system (Application Biosystems, CA, USA). β-actin was used as the internal control, and relative expression was calculated by $2^{-\Delta\Delta CT}$ method.

CRT immunofluorescence stainning

Six groups were set, *i.e.*, G1: control (PBS), G2: FRMON(US); G3: ICG@FRMON, G4: ICG@FRMON(US); G5: ICG/PDE5i@FRMON and G6: ICG/PDE5i@FRMON(US). Afterwards, corresponding treatments in G1-G6 (dose: 100 μg/mL FRMON, ICG: 4.86 μg/mL, PDE5i: 7.11 μg/mL) for 8 h were implemented and then SDT was performed in each group (US irradiations parameters were set as follows: power density = 1 W/cm^2, duty cycle = 50%, transducer = 1 MHz, duration =1 min, agar gel with a thickness of 0.5 cm as placed between the plates and ultrasound probe). After incubation for another 24 h, cells were fixed with 4% paraformaldehyde for 15 min,

and then permeabilized with 0.5% TritonX-100 at room temperature for 0.5 h. After blockading with 1% BSA for 30 min, the cells were incubated with anti-CRT dilution (AF1666, Beyotime, China) overnight at 4 °C, and then the secondary antibodies were added to incubate for another 2 h in the darkness. Finally, the nuclei were re-stained with DAPI (5 μg/mL) for 15 min and observed on LCSM.

Determination of HMGB1 in cellular supernatant

Six groups were set, *i.e.*, G1: control (PBS), G2: FRMON(US); G3: ICG@FRMON, G4: ICG@FRMON(US); G5: ICG/PDE5i@FRMON and G6: ICG/PDE5i@FRMON(US). 4T1 cells in logarithmic growth phase were seeded in 6-well plates at a density of 1×10^6/well and incubated overnight, Afterwards, corresponding treatments in G1-G6 (dose: 100 μg/mL FRMON, ICG: 4.86 μg/mL, PDE5i: 7.11 μg/mL) for 8 h were implemented and then SDT was performed in each group (US irradiations parameters were set as follows: power density = 1 W/cm^2, duty cycle = 50%, transducer = 1 MHz, duration =1 min, agar gel with a thickness of 0.5 cm as placed between the plates and ultrasound probe). Subsequently, the processed orifice plate was placed in an incubator and incubated for 24 h, and the cell supernatants were harvested *via* centrifugation and tested according to the instructions of HMGB1 enzyme-linked immunosorbent assay (ELISA) kit (EM0382, FineTest, China).

ATP assay

Identical grouping was made, *i.e.*, G1: control (PBS), G2: FRMON(US); G3: ICG@FRMON, G4: ICG@FRMON(US); G5: ICG/PDE5i@FRMON and G6: ICG/PDE5i@FRMON(US), where corresponding treatments in G1-G6 were carried out. US parameters were given as follows: power density = 1 W /cm^2, duty cycle = 50%, transducer = 1 MHz, duration =1 min, and agar gel with a

thickness of 0.5 cm was placed between the plates and ultrasound probe. In detail, treated cells were lysed at 4 ℃. Subsequently, 100 μL of ATP detection working solution (S0026, Beyotime, China) was added to the test well in advance and leaved it at room temperature for 5 minutes to make all background ATP consumed. After the sample or standard substance (20 μl) was added into the test well, the RLU value was immediately measured by multi-function microplate analyzer.

***In vitro* DCs maturation test**

Identical grouping was made, *i.e.*, G1: control (PBS), G2: FRMON(US); G3: ICG@FRMON, G4: ICG@FRMON(US); G5: ICG/PDE5i@FRMON and G6: ICG/PDE5i@FRMON(US), where corresponding treatments in G1-G6 were carried out. US parameters were given as follows: power density = 1 W /cm^2, duty cycle = 50%, transducer = 1 MHz, duration =1 min, and agar gel with a thickness of 0.5 cm was placed between the plates and ultrasound probe. In brief, immature DCs (iDCs) were extracted from the bone marrow of the 8-week-old BALB/c mice and inoculated with the pretreated 4T1 cells in 12-well plates. After co-cultivation with iDCs for 24 h, the iDCs were then stained with anti-MHCII-FITC, anti-CD11c-APC, anti-CD80-PE, and anti-CD86-PE-Cy5.5 (eBioscience, US) and examined by flow cytometry (CytoFLEX, Beckman Coulter, China). Meanwhile, the concentrations of IL-6 and TNF-α in cell supernatants were detected by ELISA kit (Neobioscience, China).

***In vivo* ICD inspection and immunosuppressive tumor microenvironment mitigation**

Female BALB/C mice aged 4 weeks were randomly divided into six groups (n=4), *i.e.*, G1: control (PBS), G2: FRMON(US); G3: ICG@FRMON, G4: ICG@FRMON(US); G5: ICG/PDE5i@FRMON and G6: ICG/PDE5i@FRMON(US). 4T1 cells (1×10^6) were injected into the right upper limb, labeled -7th day. On the -1st day, mice in different groups were treated and injected with

corresponding samples in G1-G6 through the tail vein (200 μL, dose: FRMON: 50 mg/kg, ICG: 2.43 mg/kg, PDE5i: 3.56 mg/kg). On 0 day, ultrasound irradiation was performed, where ultrasound parameters were set as follows: power density = 1 W/cm^2, duty cycle = 50%, transducer = 1 MHz, duration =15 min with 1 min invertal between two 5 min-irradiations. On the 3rd day, the lymph nodes of mice were collected and digested into single-cell suspension and subsequently analyzed by FCM. At the same time, the tumor tissues experienced IF staining, accompanied with which the serum samples of mice were detected by ELISA.

***In vivo* and *ex vivo* animal fluorescence imaging**

When the tumors reached 200 mm^3, 4T1 tumor-bearing BALB/c mice after anesthesia with isoflurane were intravenously injected with ICG/PDE5i@FRMON (200 μL, FRMON: 50 mg/kg, ICG: 2.43 mg/kg, PDE5i: 3.56 mg/kg) *via* tail vein. Subsequently, the mice were positioned with the tumor residing at the center of the scanning horizon and fluorescence images at 0 h, 2 h, 4 h, 8 h, 16 h, 24 h and 36 h were captured, respectively. After the last post-treatment, mice were sacrificed and tumor as well as some main organs (*e.g.*, heart, liver, spleen, lung and kidney) were collected to observe the ex vivo distribution of nanoparticles in tumor and normal organs on *in vivo* fluorescence instrument (PIXIS 1024, Princeton, USA) with Ex/Em: 800 nm / 850 nm. The confinement effect of mesoporous channels also enabled ICG uniform distribution for improving signal intensity for tracking tumor site, akin to viscosity-mediated confinement.[3]

Sequencing

Female BALB/c mice aged 4 weeks were randomly divided into three groups (n=3): Control (PBS), ICG@FRMON(US) and ICG/PDE5i@FRMON(US). A total of 1× 10^6 4T1 cells were injected into the right upper limb, labeled the -7th day. On the -1st day, mice in different groups were treated and

injected with corresponding samples in the three groups through the tail vein (200 μL, FRMON: 50 mg/kg, ICG: 2.43 mg/kg, PDE5i: 3.56 mg/kg). On the 0 day, ultrasound irradiation was performed, where ultrasound parameters were set as follows: power density = 1 W/cm^2, duty cycle = 50%, transducer = 1 MHz, duration =15 min with 1 min interval between two 5 min-irradiations. On the 3rd day, the tumor tissues were isolated and removed, and RNA Later prepared in advance was added within 30 min *in vitro*, followed by sequencing analysis.

***In vivo* anti-tumor and vascular normalization**

Female BALB/C mice aged 4 weeks were randomly divided to six groups (n=6), *i.e.*, G1: control (PBS), G2: FRMON(US), G3: ICG@FRMON, G4: ICG@FRMON(US), G5: ICG/PDE5i@FRMON and G6: ICG/PDE5i@FRMON(US). On the -7th day, 4T1 cells (1×10^6) were inoculated subcutaneously into the right upper limb of mice to establish subcutaneous breast cancer model. On the -1st day, mice in different groups were treated with the corresponding samples through the tail vein (200 μL, FRMON: 50 mg/kg, ICG: 2.43 mg/kg, PDE5i: 3.56 mg/kg), and ultrasound irradiations were performed on the 0, 2 and 4 days, respectively. Ultrasound parameters were set as follows: power density = 1 W/cm^2, duty cycle = 50%, transducer = 1 MHz, duration =15 min with 1 min interval between two 5 min-irradiations. Temperature, body weight and tumor volume (length × $width^2/2$) of mice were measured every two days. On the 0 d, 7 d, and 14 d, respectively, the saturation of pulse oximetry (SpO_2) in tumor was detected on the clinical pulse oximeter (Beneview T1, Mindray). On the 16th day, the mice were sacrificed, and the tumor tissues were harvested and analyzed by ELISA, IHC and IF staining and FCM analysis, respectively. Meanwhile, the main organs of the mice were sectioned and stained by H&E.

As well, another 30 mice were divided into the same groups (n=5) as that mentioned above and

received the corresponding treatments for survival analysis observation. Notably, as the tumor volume reached up to 2000 mm^3 or premature death of mice occurred, this time was designed as the end point.

Immunosuppressive tumor microenvironment (ITM) mitigation against tumor metastasis in ICB test

Herein, bilaterally-inoculated 4T1 tumor model with lung metastasis were established, and immune checkpoint blockade (ICB) therapy was combined to further verify ITM mitigation or reversion *via* evaluating the anti-tumor and anti-metastasis outcome. Female BALB/C mice aged 4 weeks were divided into the following groups (n=5): Control (PBS), ICG/PDE5i@FRMON(US), αPDL1, αPDL1+ICG/PDE5i@FRMON(US). Akin to above model establishment, the left subcutaneous breast cancer model was established on the -7th day. On the -2nd day, distant subcutaneous tumor called as metastasized nodule was established on the right lower limb of mice after injecting 4T1 cells (1×10^6). As well, the breast cancer lung metastasis model was established by injecting 4T1 cells (1×10^5) through the tail vein. On -1st day, all mice were treated with corresponding samples (200 μL, FRMON: 50 mg/kg, ICG: 2.43 mg/kg, PDE5i: 3.56 mg/kg) and on the 0 d, 2 d and 4 d, ultrasound irradiations were performed. Ultrasound parameters were set as follows: power density = 1 W/cm^2, duty cycle = 50%, transducer = 1 MHz, duration =15 min with 1 min interval between two 5 min-irradiations. The mice were injected with α-PDL1 (100 μg per mouse) on the 1 d, 3 d and 5 d. Observation was performed once per 7 days. After 21 days, mice were sacrificed, and the distant tumor tissue on the right side was acquired for ELISA detection, and the subcutaneous tumor on the right side was subjected to immunofluorescence staining. The lung tissues were sectioned and stained with HE immunohistochemical staining to observe the metastasis. Serum was collected and

detected by ELISA. Lymphocytes in spleen were detected by flow cytometry.

ITM mitigation and vascular normalization for favoring CAR-T immunotherapy

NKG2D target-engineered CAR-T cells were purchased from GENECHEM Co. (Shanghai, China). Female NSG mice aged 4 weeks were divided into the following groups (n=5): G1: control (PBS), G2: FRMON(US); G3: ICG@FRMON; G4: ICG@FRMON(US); G5: ICG/PDE5i@FRMON and G6: ICG/PDE5i@FRMON(US); G7: CAR-T; G8: ICG/PDE5i@FRMON(US)+CAR-T, where ICD/PDE5i@FRMON(US) represent SDT. Ultrasound parameters were set as follows: power density = 1 W/cm^2, duty cycle = 50%, transducer = 1 MHz, duration =15 min with 1 min interval between two 5 min-irradiations. Firstly, the MDA-MB-231 tumor-bearing NSG mice models were successfully established through injecting 2×10^6 cells into the leg of mice. After reaching 0.5 cm, the corresponding samples in each group were administered (200 μL, FRMON: 50 mg/kg, ICG: 2.43 mg/kg, PDE5i: 3.56 mg/kg) per day before SDT, and after 2 h post-SDT, 1×10^7 CAR-T cells were injected, during which the state of mice was monitored once per 5 days. On the 25th day, the tumor tissue was dissected and detected by H&E staining, immunofluorescence, ELISA and FCM analysis. As well, another 40 mice were divided into the same groups (n=5) as mentioned above and received the corresponding treatments for survival analysis. Notably, as the tumor volume reached up to 1000 mm^3 or premature death of mice occurred, this time was designed as the end point.

Biosafety evaluations

Female BALB/C mice aged 4 weeks were divided into four groups (n=3), *i.e.*, control (PBS), FRMON (50 mg/kg), FRMON (100 mg/kg) and FRMON (200 mg/kg). The body weight and temperature of the mice were monitored every 10 days. After 30 days, blood was collected from the eyeballs, followed by blood biochemical tests and liver & kidney function tests. Organs were stained

with HE and photographed for observation.

Statistics

All statistics are performed with SPSS 22.0 (IBM Corporation, USA). The picture was drawn using GraphPad Prism 8.0 (San Diego, CA). The numerical results were expressed as mean ± standard deviations (SD). Statistical significance between the groups was determined by *t*-test or one-way analysis of variance (ANOVA). Statistical significance was set at a P-value < 0.05, where $*P < 0.05$, $** P < 0.01$, and $*** P < 0.001$.

References

[1] K. Zhang, H. Chen, Y. Zheng, Y. Chen, M. Ma, X. Wang, L. Wang, D. Zeng, J. Shi, *J. Mater. Chem.* **2012**, *22*, 12553-12561.

[2] K. Zhang, H. Chen, P. Li, X. Bo, X. Li, Z. Zeng, H. Xu, *ACS Appl. Mater. Interfaces* **2015**, *7*, 18590-18599.

[3] K. Zhang, H.-Y. Li, J.-Y. Lang, X.-T. Li, W.-W. Yue, Y.-F. Yin, D. Du, Y. Fang, H. Wu, Y.-X. Zhao, C. Xu, *Adv. Funct. Mater.* **2019**, *29*, 1905124.

Supplementary Figures

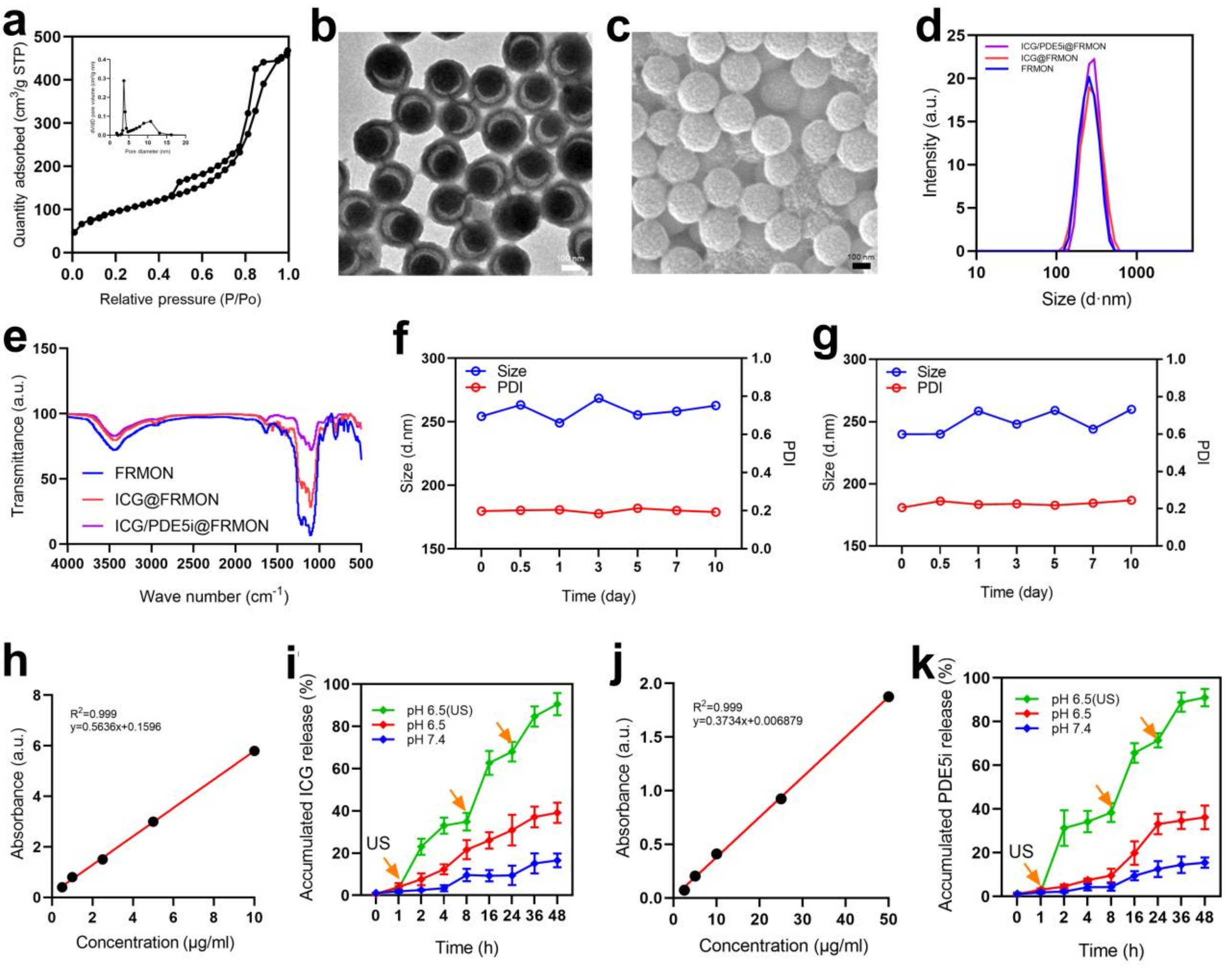

Figure S1. Characterizations and evaluations of FRMON and ICG/PDE5i@FRMON. (a) N_2 adsorption and desorption isotherm and pore diameter distribution of FRMON carriers. (b,c) TEM (b) and SEM (c) images of ICG/PDE5i@FRMON. (d) Particle size distributions of FRMON, ICG@FRMON and ICG/PDE5i@FRMON *via* DLS. (e) Fourier transform infrared (FTIR) spectra of FRMON, ICG@FRMON and ICG/PDE5i@FRMON. (f,g) Stability tests of ICG/PDE5i@FRMON in PBS (f) and FBS (g) via evaluating its particle size and polydispersity index (PDI) using DLS. (h) Standard curve of ICG obtained according to the linear relationship of ICG concentration to absorbance intensity at 780 nm. (i) Time-dependent ICG release from ICG/PDE5i@FRMON at different pH values (6.5 and 7.4), and especially ultrasound (US) irradiations were implemented in the group (pH=6.5) at 1 h, 8 h and 24 h, respectively, as indicated by saffron yellow arrows. (j) Standard curve of PDE5i obtained according to the linear relationship of PDE5i concentration to absorbance intensity at 290 nm. (k) Time-dependent PDE5i release from ICG/PDE5i@FRMON at different pH values (6.5 and 7.4), and especially US irradiations were implemented in the group (pH=6.5) at 1 h, 8 h and 24 h, respectively, as indicated by saffron yellow arrows. Data are expressed as mean±SD (n=3).

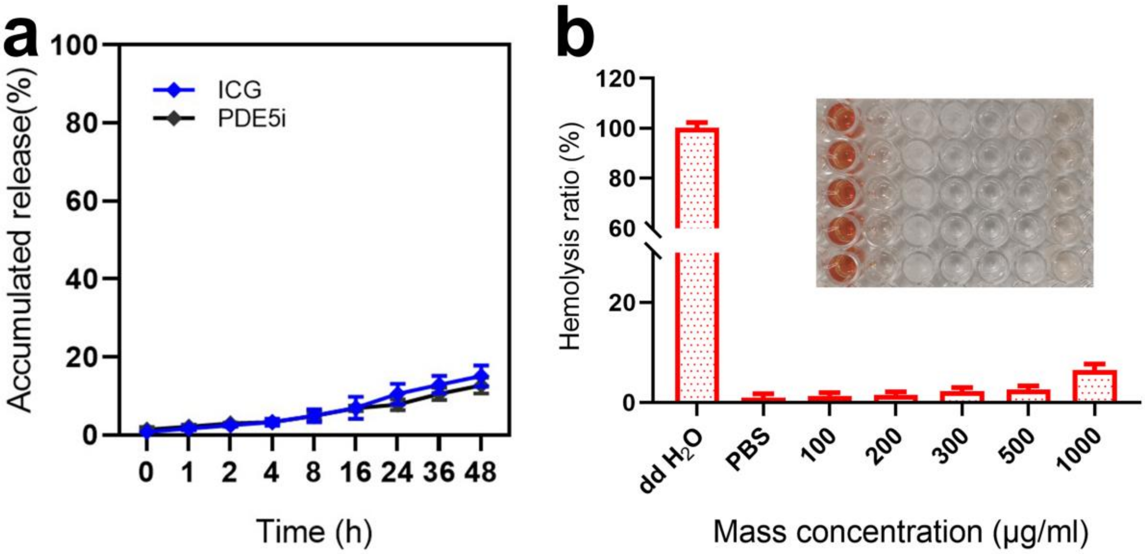

Figure S2. (a) Time-dependent ICG and PDE5i release profiles from ICG/PDE5i@FRMON in FBS. Data are expressed as mean ± SD (n=3). (b) Hemolysis ratios of red cells in dd H_2O, PBS and ICG/PDE5i@FRMON nanoplatforms with varied concentrations (100 μg/mL, 200 μg/mL, 300 μg/mL, 500 μg/mL and 1000 μg/mL); Data are expressed as mean±SD (n=5).

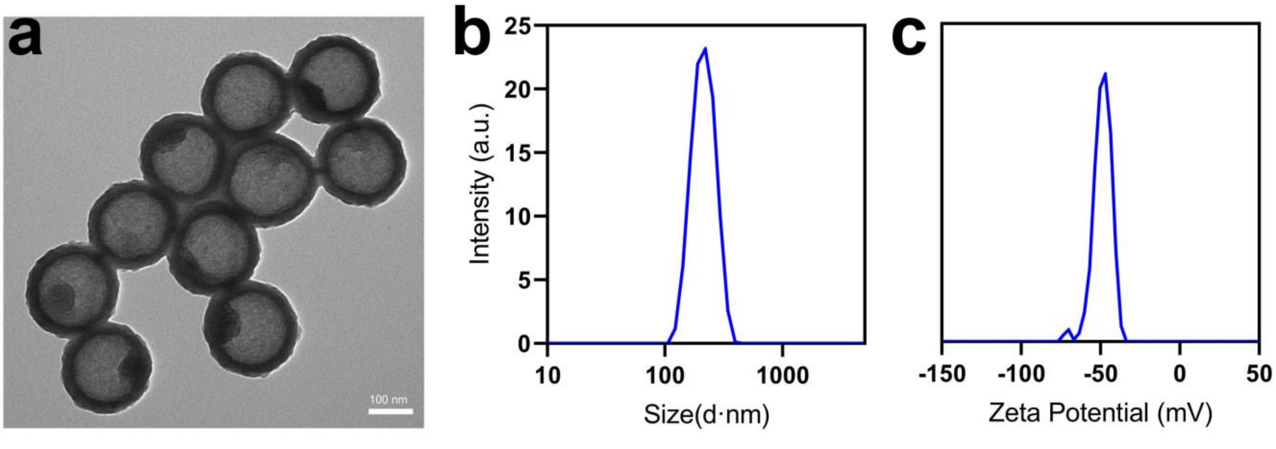

Figure S3. Characterizations of FHMON carriers. (a) TEM image of FHMON carriers. (b,c) Particle size distribution (b) and zeta potential (c) of FHMON carriers.

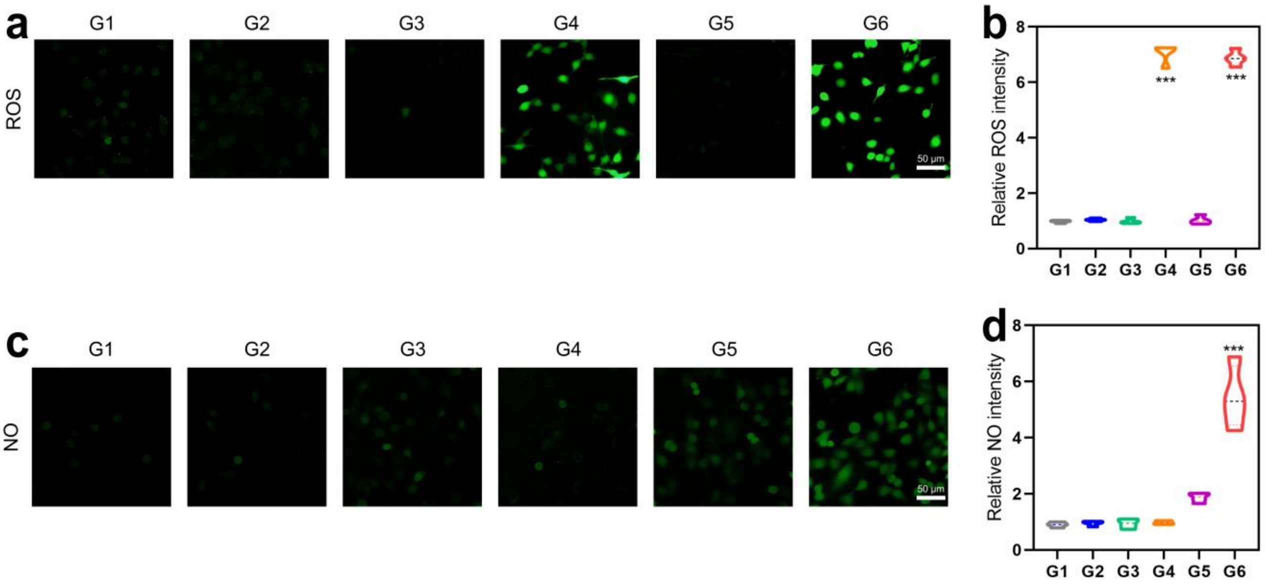

Figure S4. (a,b) LCSM images (a) and quantitative data (b) of MDA-MB-231 cells stained with ROS indicator (*i.e.*, DCFH-DA) after corresponding treatment in different groups (G1-G6) for evaluating ROS level. (c,d) LCSM images (c) and quantitative data (d) of MDA-MB-231 cells stained with NO probe (*i.e.*, DAF-FM DA) after corresponding treatment in different groups (G1-G6) for evaluating NO level. G1-G6 represent control (PBS), FRMON(US), ICG@FRMON, ICG@FRMON(US), ICG/PDE5i@FRMON and ICG/PDE5i@FRMON(US), respectively; and Scale bar: 50 μm. Data are expressed as mean ± SD (n=4). Statistical significance was determined by ANOVA, and P*** < 0.001.

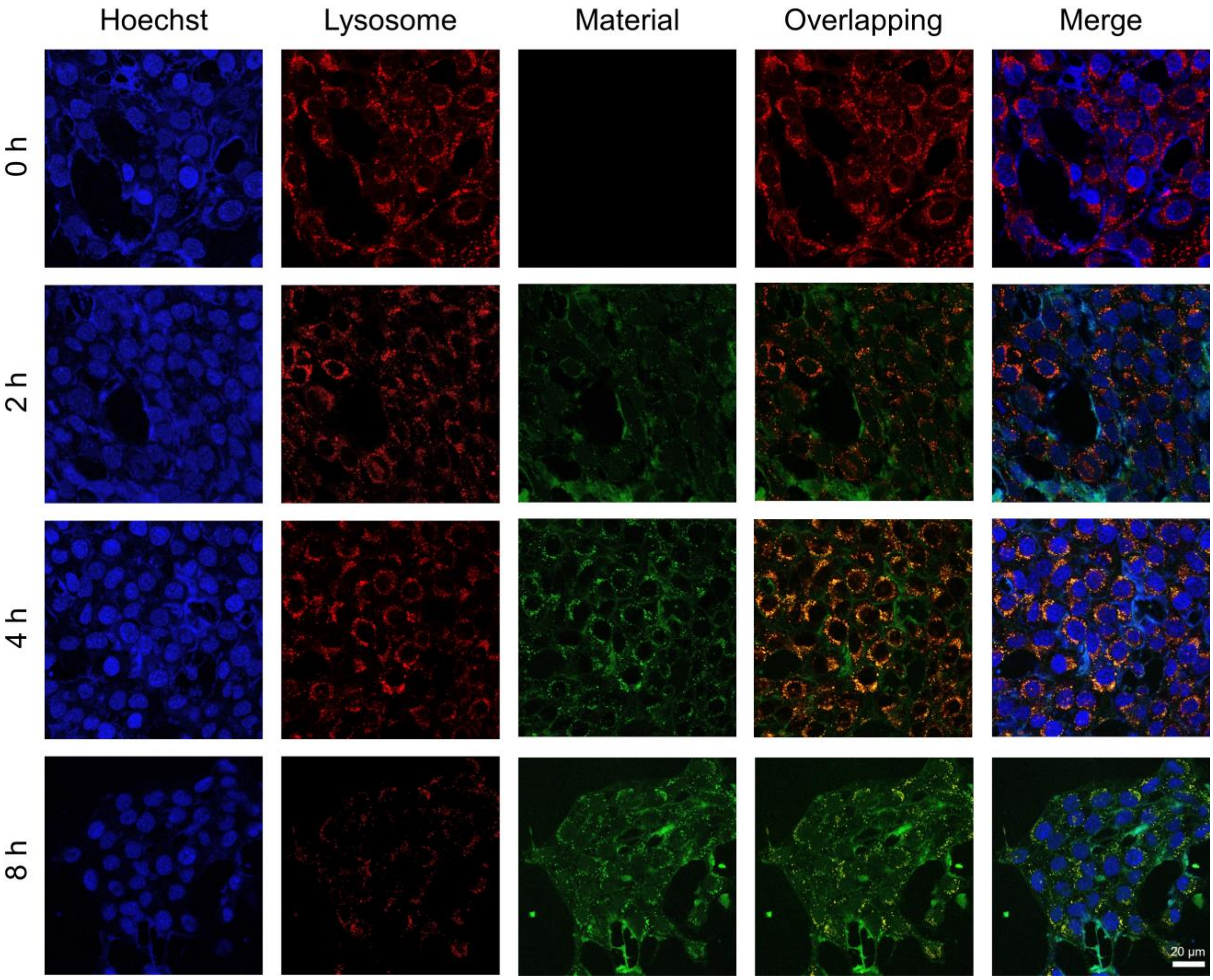

Figure S5. LCSM images of 4T1 after incubations with ICG/PDE5i@FRMON for different time periods to trace the endosomal escape, where blue, red and green colours represent cell nuclei, lysosome and material, respectively. At 8 h, endosomal escape is clearly found due to lysosome rupture; and Scale bar: 20 μm..

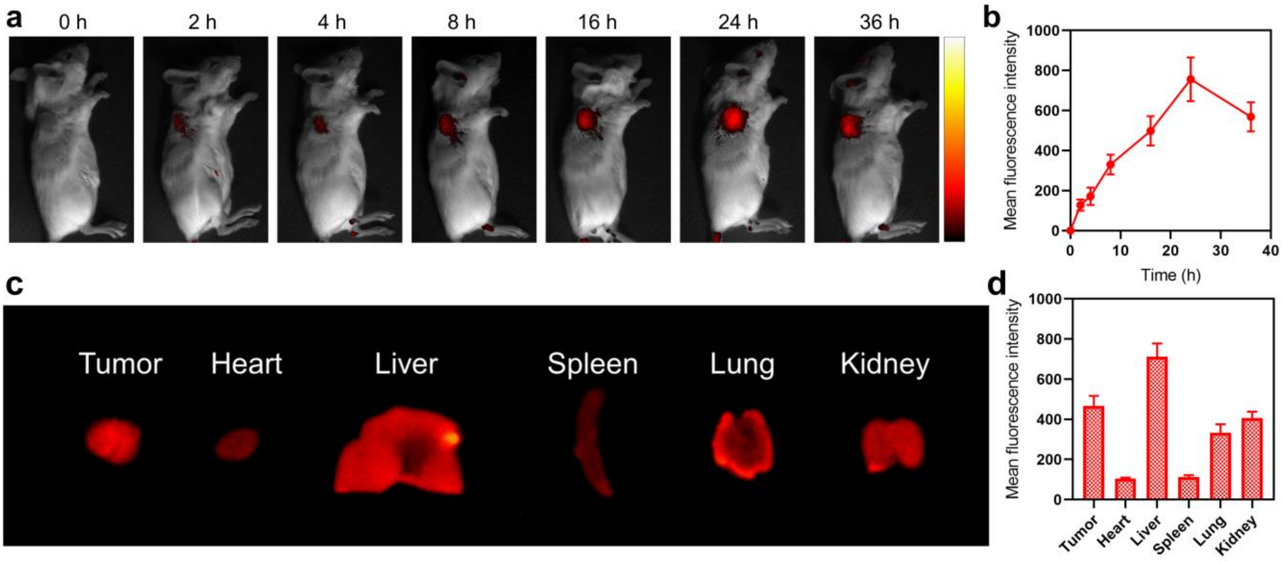

Figure S6. (a,b) *In vivo* fluorescence images (a) and mean fluorescence intensity (b) of 4T1 tumors implanted on mice after injecting ICG/PDE5i@FRMON as a function of time; (c,d) *Ex vivo* fluorescence images (c) and mean fluorescence intensity (d) of normal organs and tumor harvested from 4T1 tumor-bearing mice after 36 h post-injection of ICG/PDE5i@FRMON. Data are expressed as mean ± SD (n=3).

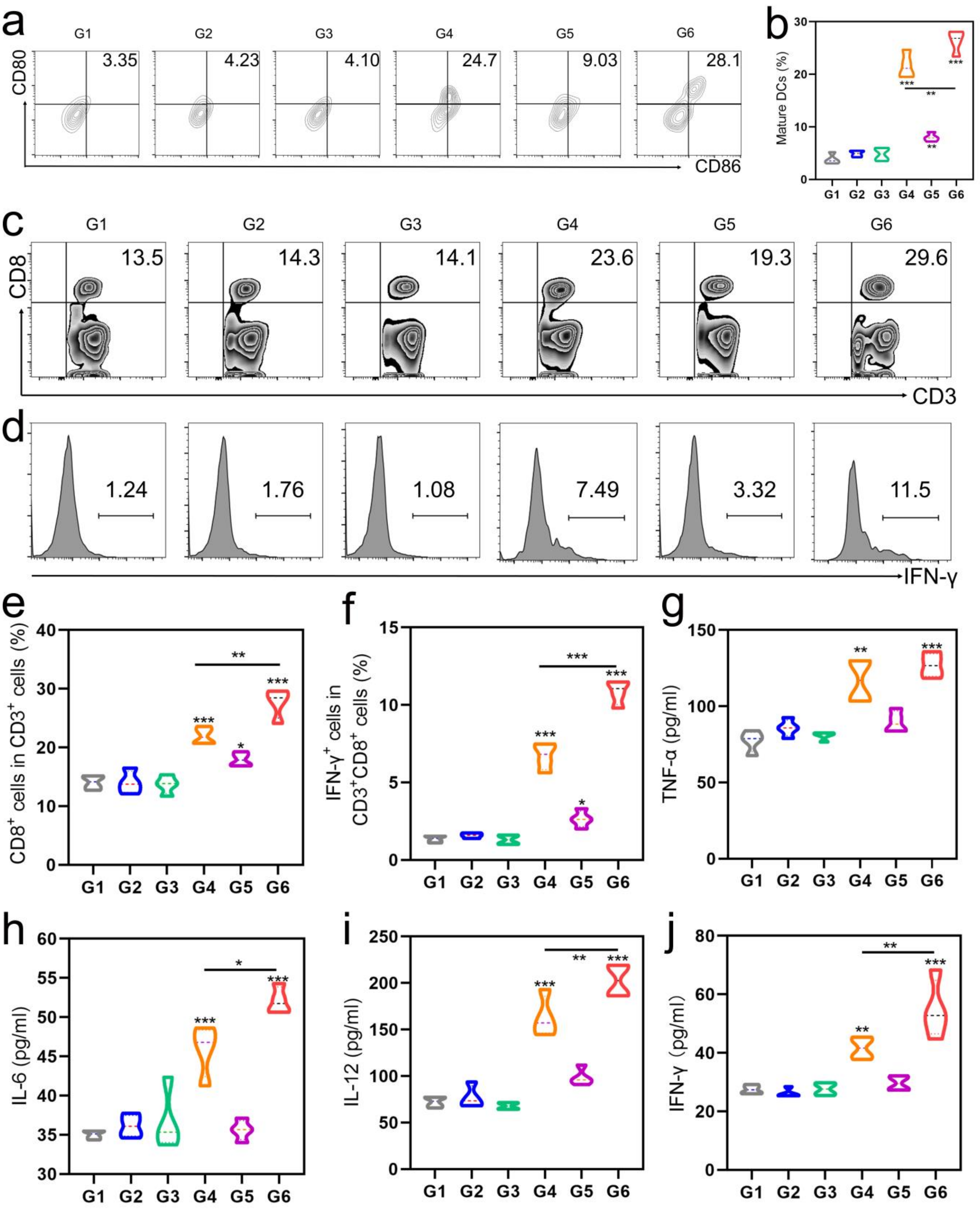

Figure S7. ***In vivo*** **tests on ICD potentiation and systematic immune response activations.** (a,b) FCM patterns (a) and quantitative data (b) of matured DCs (CD80+CD86+) harvested from TDLNs in 4T1 tumor-bearing mice after 3 days post-corresponding treatment in different groups (G1-G6). (c-f) FCM patterns (c,d) and quantitative percentages (e,f) of CD8+ T cells gating from CD3+ cells

(c,e) and cytotoxic T lymphocytes (CTLs, CD8+IFNγ+) gating from CD8+CD3+ cells (d,f) harvested from tumor-draining lymph nodes in 4T1 tumor-bearing mice after 3 days post-corresponding treatment in different groups (G1-G6). (g-j) Secretion levels of cytokines including TNF-α (g), IL-6 (h), IL-12 (i) and IFN-γ (j) in serum harvested from 4T1-bearing mice after 3 days post-corresponding treatment in different groups (G1-G6). Data are expressed as mean ± SD (n=4). Statistical significance between the groups was determined by one-way analysis of variance (ANOVA), and *P<0.05, **P<0.01 and ***P<0.001. G1-G6 represent control (PBS), FRMON(US); ICG@FRMON, ICG@FRMON(US), ICG/PDE5i@FRMON and ICG/PDE5i@FRMON(US), respectively.

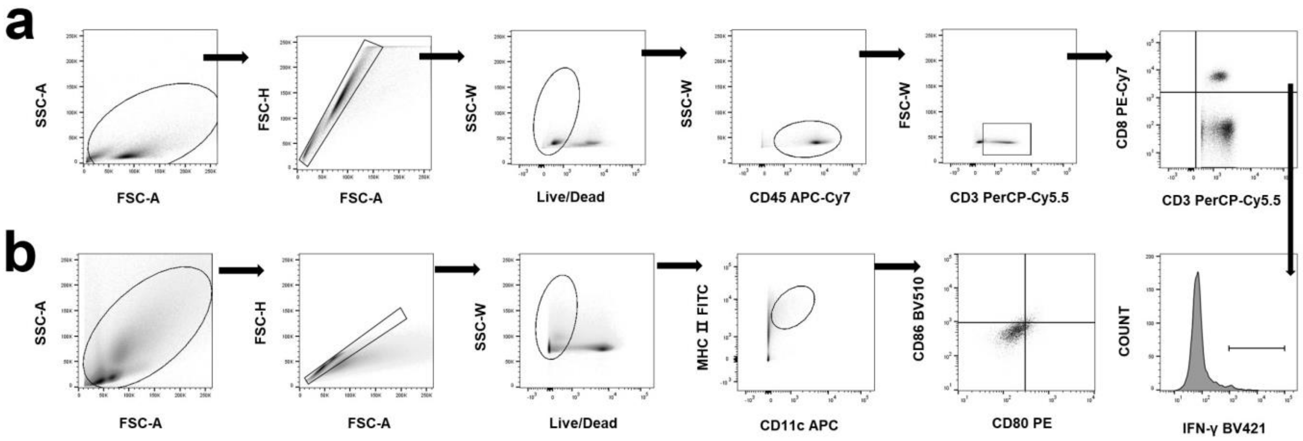

Figure S8. Representative example of scatter lots from cytometry analyses based on 5-color showing CD3+CD8+, CD8+INF-γ+ and DCs (CD80+CD86+CD11c+MHCII+) from tumor-draining lymph nodes in 3-day ICD test, respectively. Arrows indicate the gating strategy.

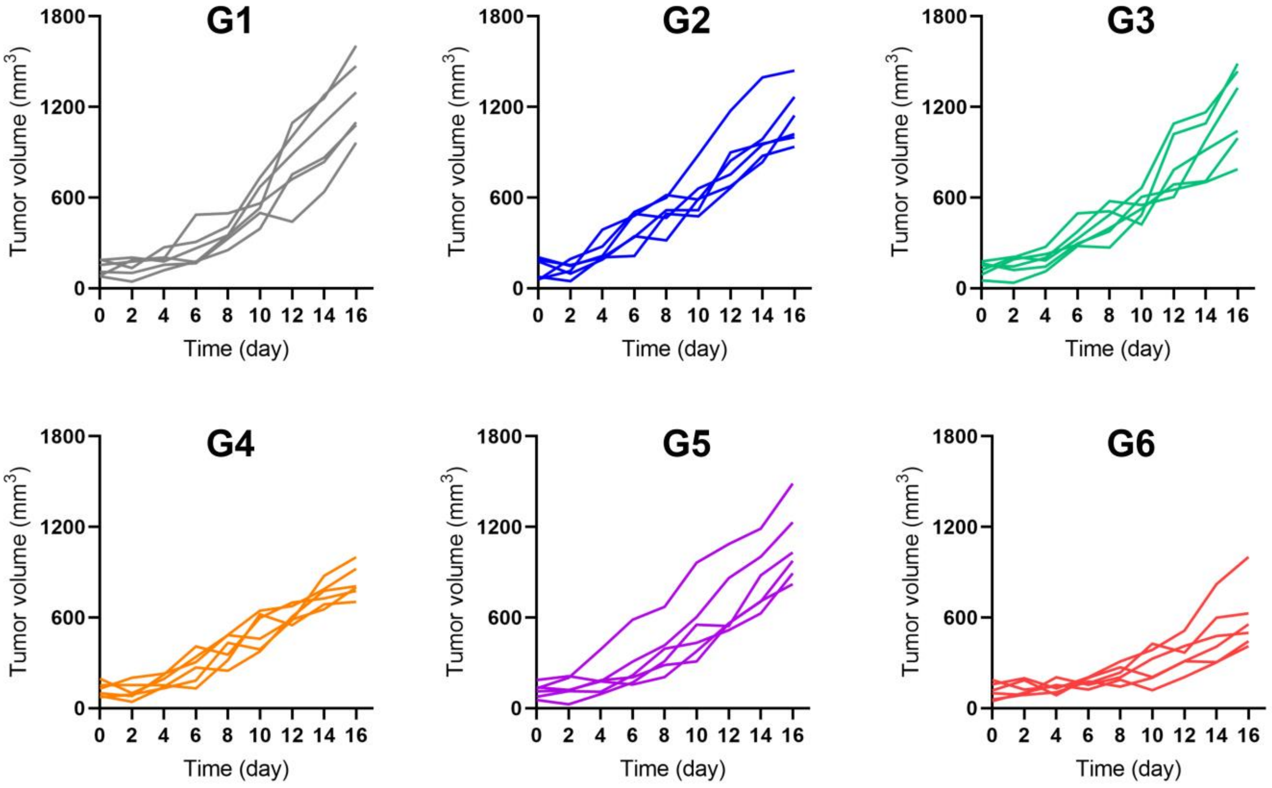

Figure S9. Time-dependent tumor growth curves of each mouse that experienced corresponding treatment in different groups (G1-G6). G1-G6 represent control (PBS), FRMON(US); ICG@FRMON, ICG@FRMON(US), ICG/PDE5i@FRMON and ICG/PDE5i@FRMON(US), respectively.

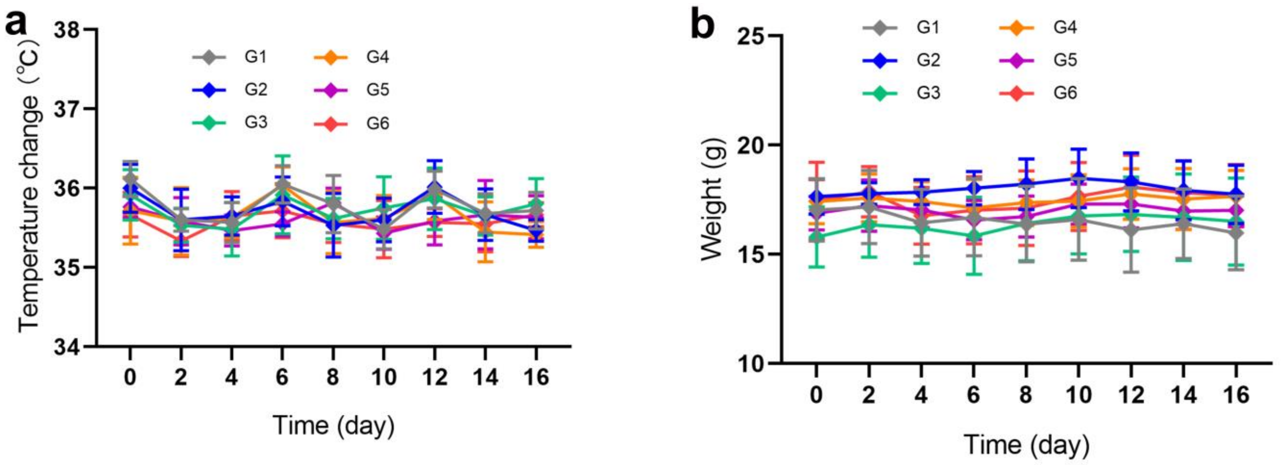

Figure S10. (a,b) Time-dependent temperature change curves (a) and weight variation (b) of 4T1-bearing mice that experienced corresponding treatment in different groups (G1-G6). Data are expressed as mean ± SD (n=6). G1-G6 represent control (PBS), FRMON(US); ICG@FRMON, ICG@FRMON(US), ICG/PDE5i@FRMON and ICG/PDE5i@FRMON(US), respectively.

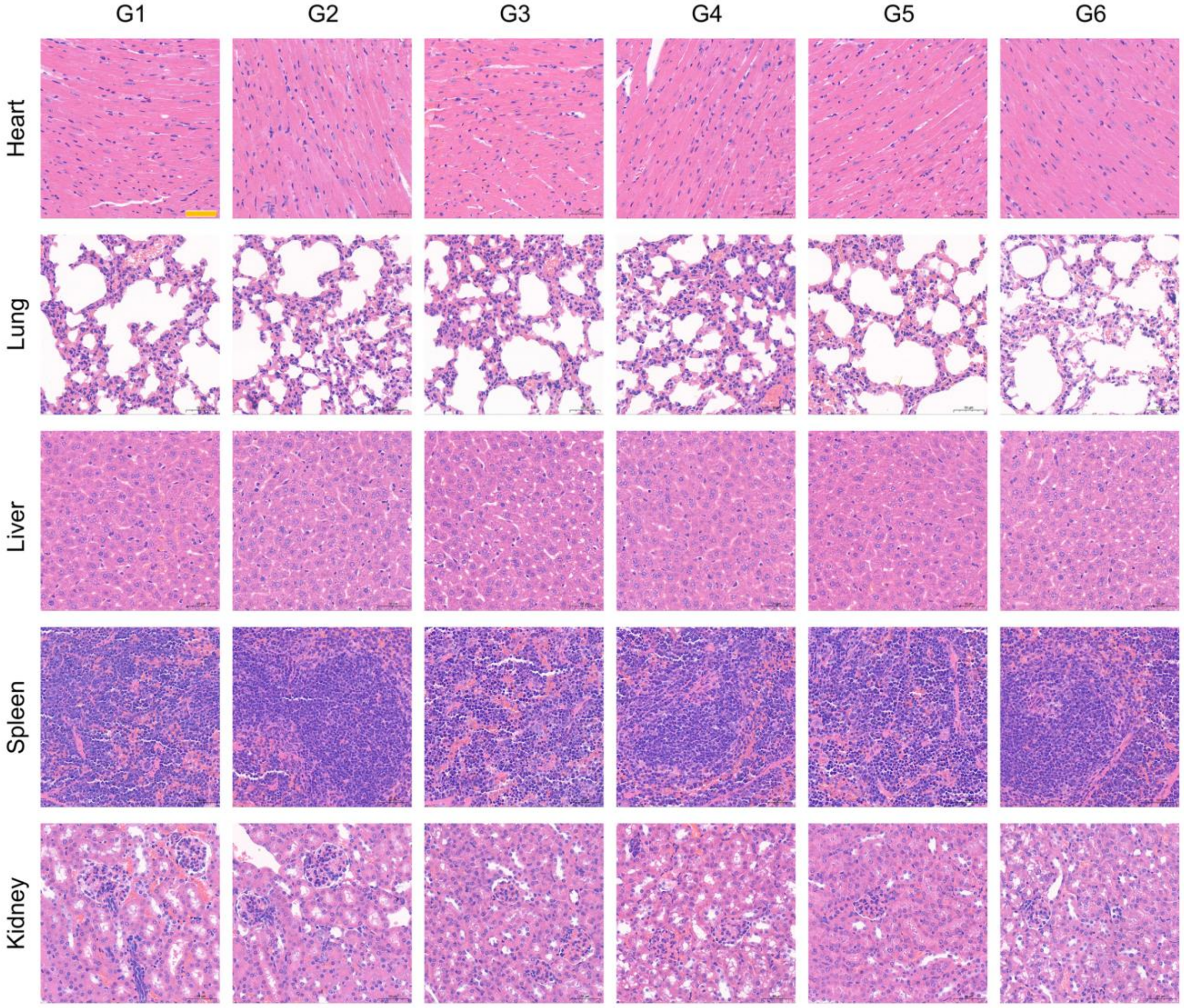

Figure S11. Optical microscopic HE-stained images of normal organs harvested from 4T1-bearing mice that experienced corresponding treatment in different groups (G1-G6) at the end of experimental period (16 days). G1-G6 represent control (PBS), FRMON(US); ICG@FRMON, ICG@FRMON(US), ICG/PDE5i@FRMON and ICG/PDE5i@FRMON(US), respectively; and Scale bar: 50 μm.

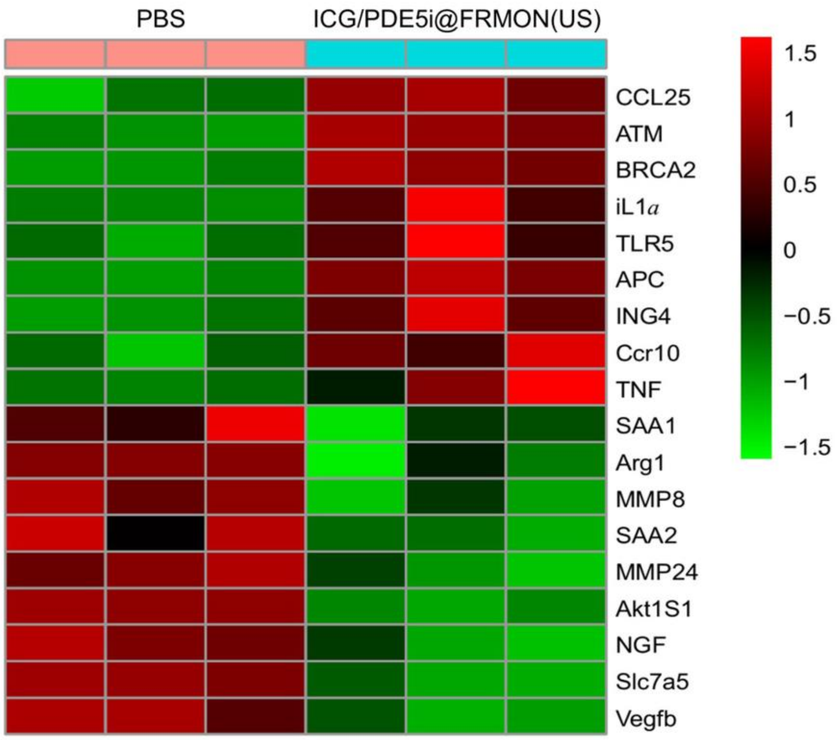

Figure S12. Heatmap for determining the total differential genes of tumors between PBS and ICG/PDE5i@FRMON(US).

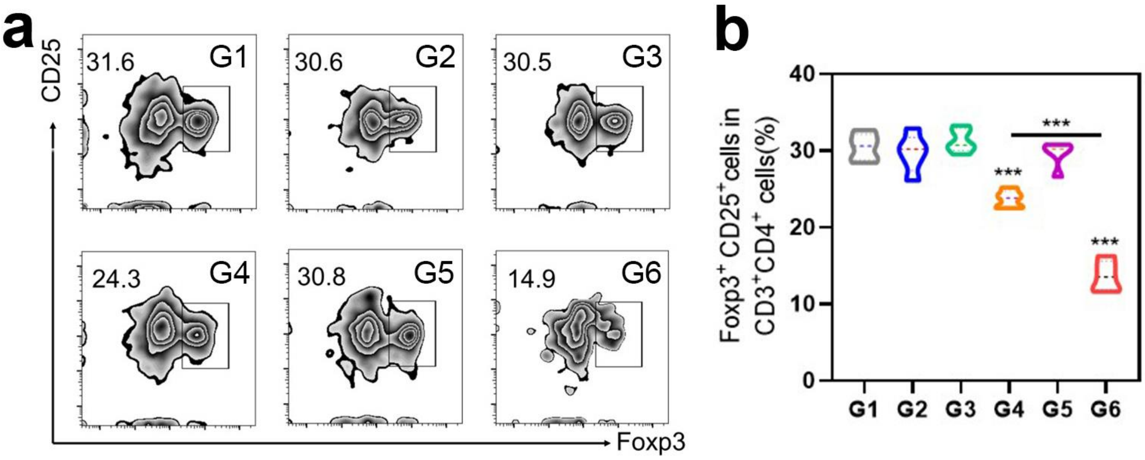

Figure S13. FCM patterns (a) and quantitative percentages (b) of FOXP3+CD4+CD25+ (Tregs) in 4T1 tumors after corresponding treatment in different groups (G1-G6). Data are expressed as mean ± SD (n=5). Statistical significance was determined by ANOVA, and ***P<0.001. Note, G1-G6 represent control (PBS), FRMON(US), ICG@FRMON, ICG@FRMON(US), ICG/PDE5i@FRMON and ICG/PDE5i@FRMON(US), respectively.

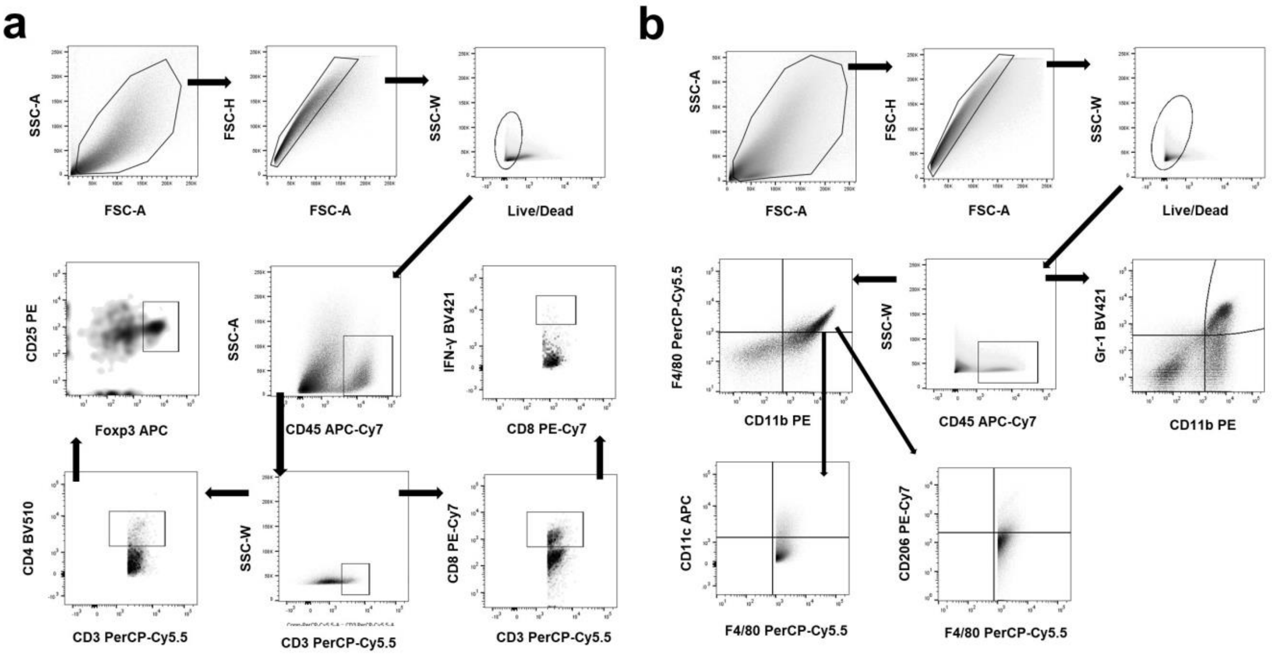

Figure S14. (a) Representative example of scatter lots from cytometry analyses based on 8- color showing Tregs (FOXP3+CD25+CD4+CD3+) and effector T cells (INF-γ+CD8+CD3+) from a tumor. Arrows indicate the gating strategy. (b) Representative example of scatter lots from cytometry analyses based on 7- color showing MDSCs (Gr-1+CD11b+CD45+), M1-type macrophages (CD11c+CD11b+F4/80+) and M2-type macrophages (CD206+CD11b+F4/80+) from a tumor in ICD-mediated anti-tumor tests, respectively. Arrows indicate the gating strategy.

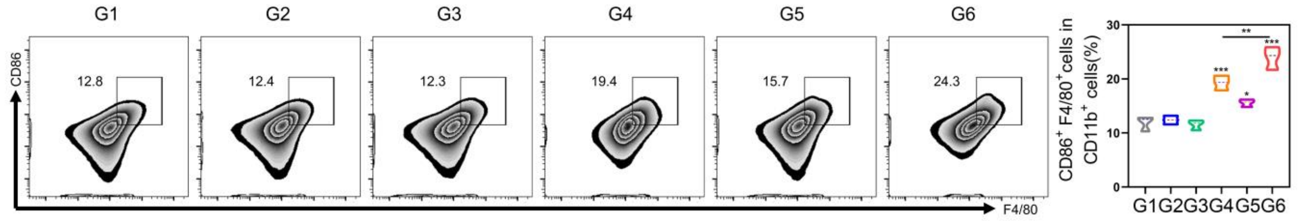

Figure S15. FCM patterns (a) and quantitative percentages (b) of M1-type macrophage (CD86+F4/80+CD11b+) in 4T1 tumors after 16 days post-corresponding treatment in different groups (G1-G6). Data are expressed as mean ± SD (n=3). Statistical significance was determined by ANOVA, and *P<0.05, **P<0.01 and ***P<0.001. Note, G1-G6 represent control (PBS), FRMON(US), ICG@FRMON, ICG@FRMON(US), ICG/PDE5i@FRMON and ICG/PDE5i@FRMON(US), respectively.

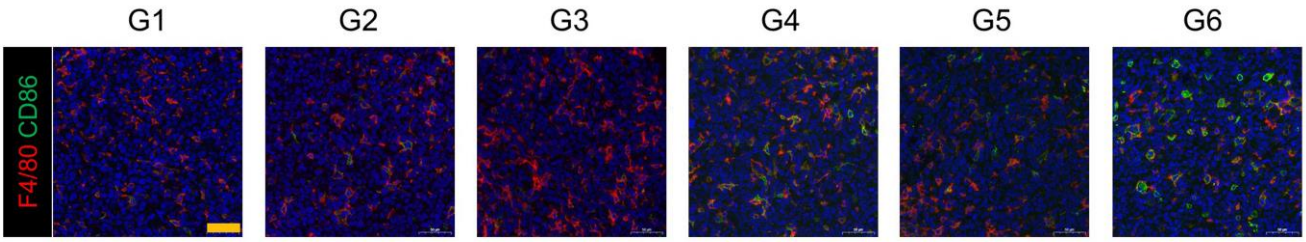

Figure S16. LCSM images of 4T1 tumor slices co-stained with F4/80 and CD86, and the tumor slices were harvested from 4T1 tumor-bearing mice that received different treatments in G1-G6. Note, G1-G6 represent control (PBS), FRMON(US), ICG@FRMON, ICG@FRMON(US), ICG/PDE5i@FRMON and ICG/PDE5i@FRMON(US), respectively; and Scale bar: 50 μm.

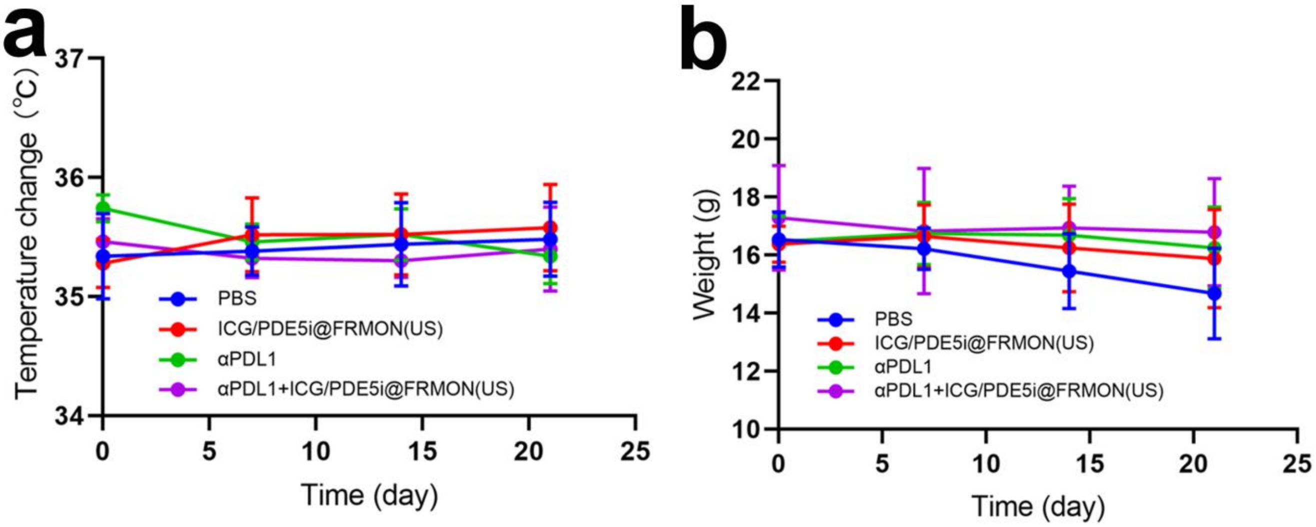

Figure S17. Time-dependent temperature change curves (a) and weight variation (b) of bilaterally-implanted 4T1 tumor and lung metastasis-bearing mice that experienced corresponding treatment in different groups, i.e., PBS, ICG/PDE5i@FRMON(US), aPD-L1 and aPD-L1+ ICG/PDE5i@FRMON(US). Data are expressed as mean ± SD (n=5).

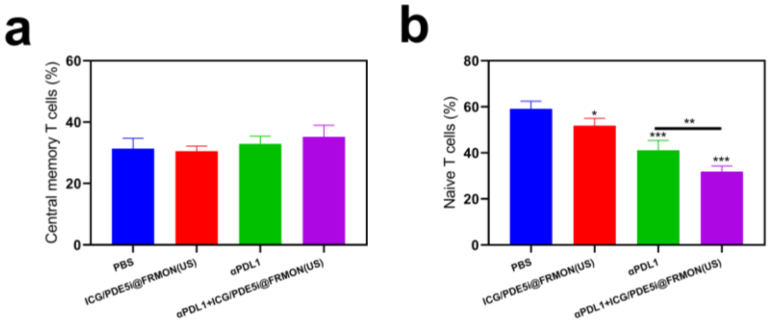

Figure S18. Quantitative percentages of (a) CD44+CD62L+ gating from CD8+CD3+ (Central memory T cells, Tcm) and CD44-CD62L+ gating from CD8+CD3+ (Naive T cells). Data are expressed as mean ± SD (n=5). Statistical significance was determined by ANOVA, and $^{*}P<0.05$, $^{**}P<0.01$, and $^{***}P<0.001$.

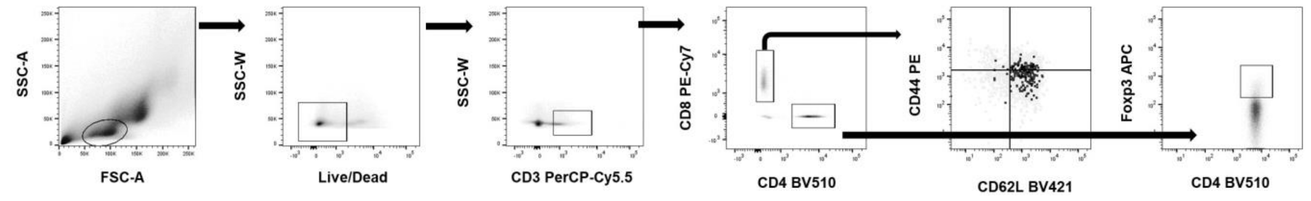

Figure S19. Representative example of scatter lots from cytometry analyses based on 7- color showing Tem (CD44+CD62L-CD8+) and Tregs (FOXP3+CD4+) from a spleen in ICB tests. Arrows indicate the gating strategy.

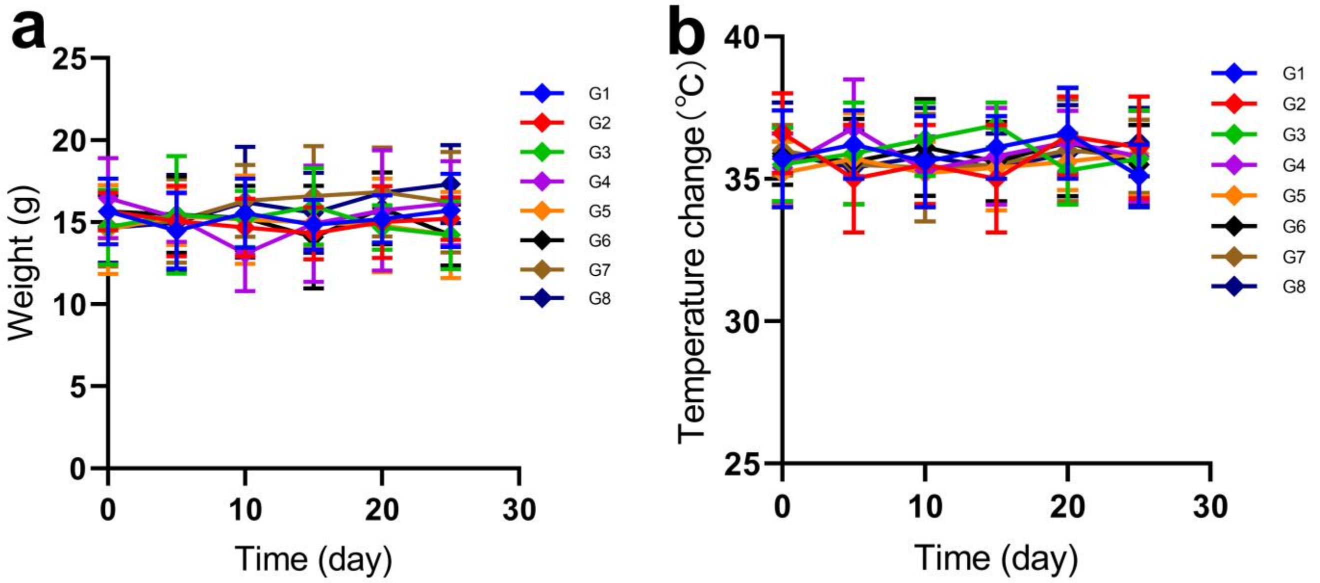

Figure S20. Time-dependent weight variation (a) and temperature change curves (b) of MDA-MB-231 tumor-bearing NSG mice that experienced corresponding treatment in different groups (G1-G8), *i.e.*, PBS, ICG/PDE5i@FRMON(US), CAR-T and CAR-T+ ICG/PDE5i@FRMON(US). Data are expressed as mean ± SD (n=5). CAR-T cells are those NKG2D target-engineered T cells. Note, G1-G8 represent PBS, FRMON (US), ICG@FRMON, ICG@FRMON (US), ICG/PDE5i@FRMON, ICG/PDE5i@FRMON(US), CAR-T and ICG/PDE5i@FRMON(US)+CAR-T+SDT, respectively, where ICG/PDE5i@FRMON(US) is also called as SDT.

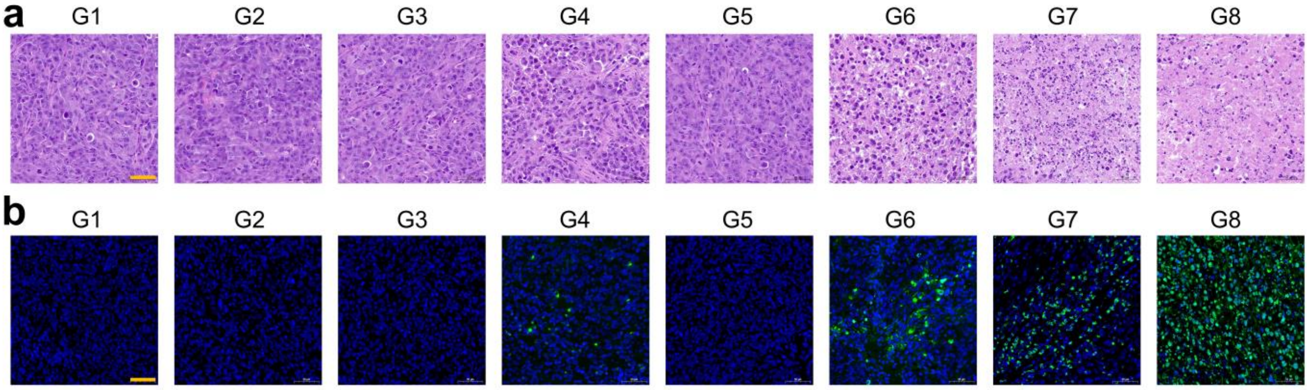

Figure S21. (a,b) Optical microscopic HE-stained images (a) and TUNEL-stained LCSM images (b) of tumor slices harvested from MDA-MB-231 tumor-bearing NSG mice that experienced corresponding treatment in different groups at the end of experimental period (25 days), where green represent apoptosis. Note, SDT means ICG/PDE5i@FRMON(US), and Scale bar: 50 μm. Note, G1-G8 represent PBS, FRMON (US), ICG@FRMON, ICG@FRMON (US), ICG/PDE5i@FRMON, ICG/PDE5i@FRMON(US), CAR-T and ICG/PDE5i@FRMON(US)+CAR-T+SDT, respectively, where ICG/PDE5i@FRMON(US) is also called as SDT.

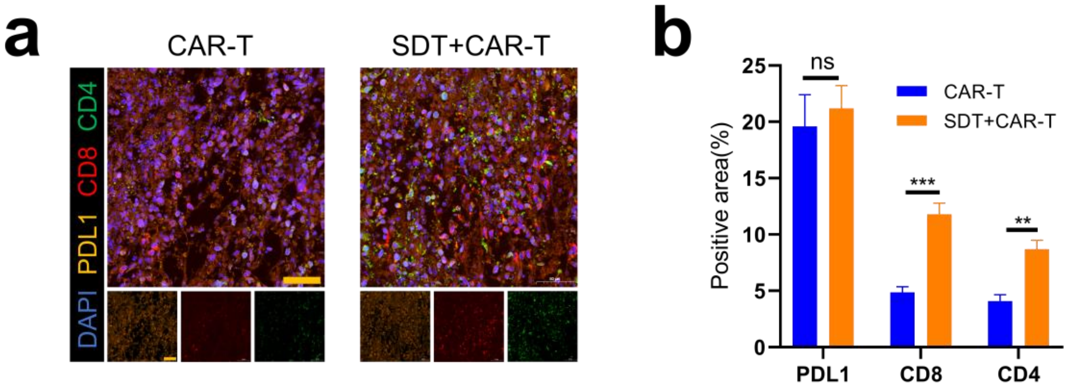

Figure S22. LCSM images of MDA-MB-231 tumor slices co-stained with DAPI, PDL1, CD8 and CD4 (a) immunofluorescence and (b) positive area in MDA-MB-231 tumor implanted on NSG mice who received the treatment with CAR-T and SDT+CAR-T, respectively, and scale bar: 50 μm. Note, SDT represents ICG/PDE5i@FRMON(US). Data are expressed as mean ± SD (n=3). Statistical significance was determined by *t*-test, and ‘ns’ means no significance, **P<0.01, and ***P<0.001.

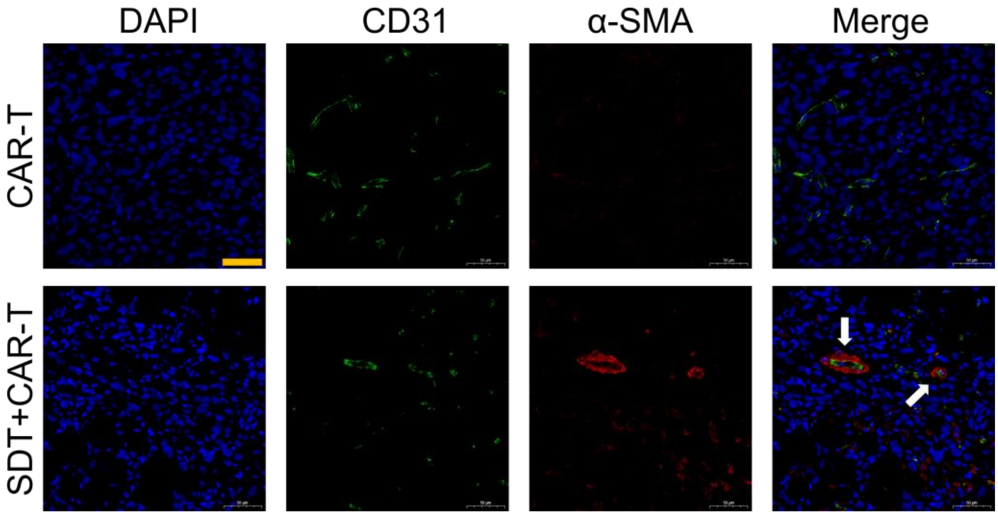

Figure S23. LCSM images of MDA-MB-231 tumor slices co-stained with DAPI, CD31 and α-SMA immunofluorescence in MDA-MB-231 tumor implanted on NSG mice who received the treatment with CAR-T and SDT+CAR-T, respectively, and scale bar: 50 μm. Note, SDT represents ICG/PDE5i@FRMON(US).

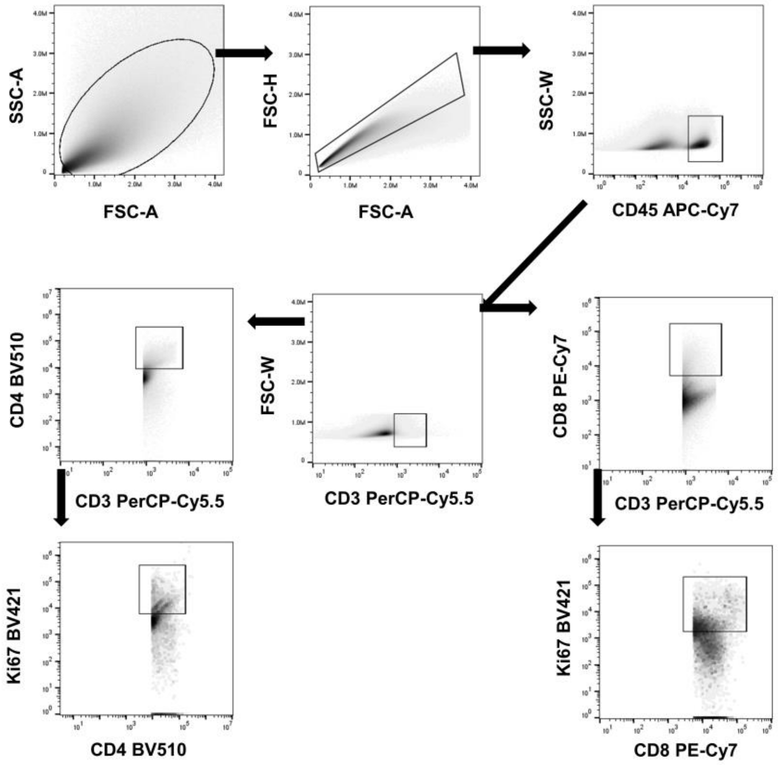

Figure S24. Representative example of scatter lots from cytometry analyses based on 5- color showing CD4+CD3+, CD8+CD3+, Ki67+CD4+CD3+ and Ki67+CD8+CD3+ from a tumor in CAR-T immunotherapy test on NSG mice. Arrows indicate the gating strategy.

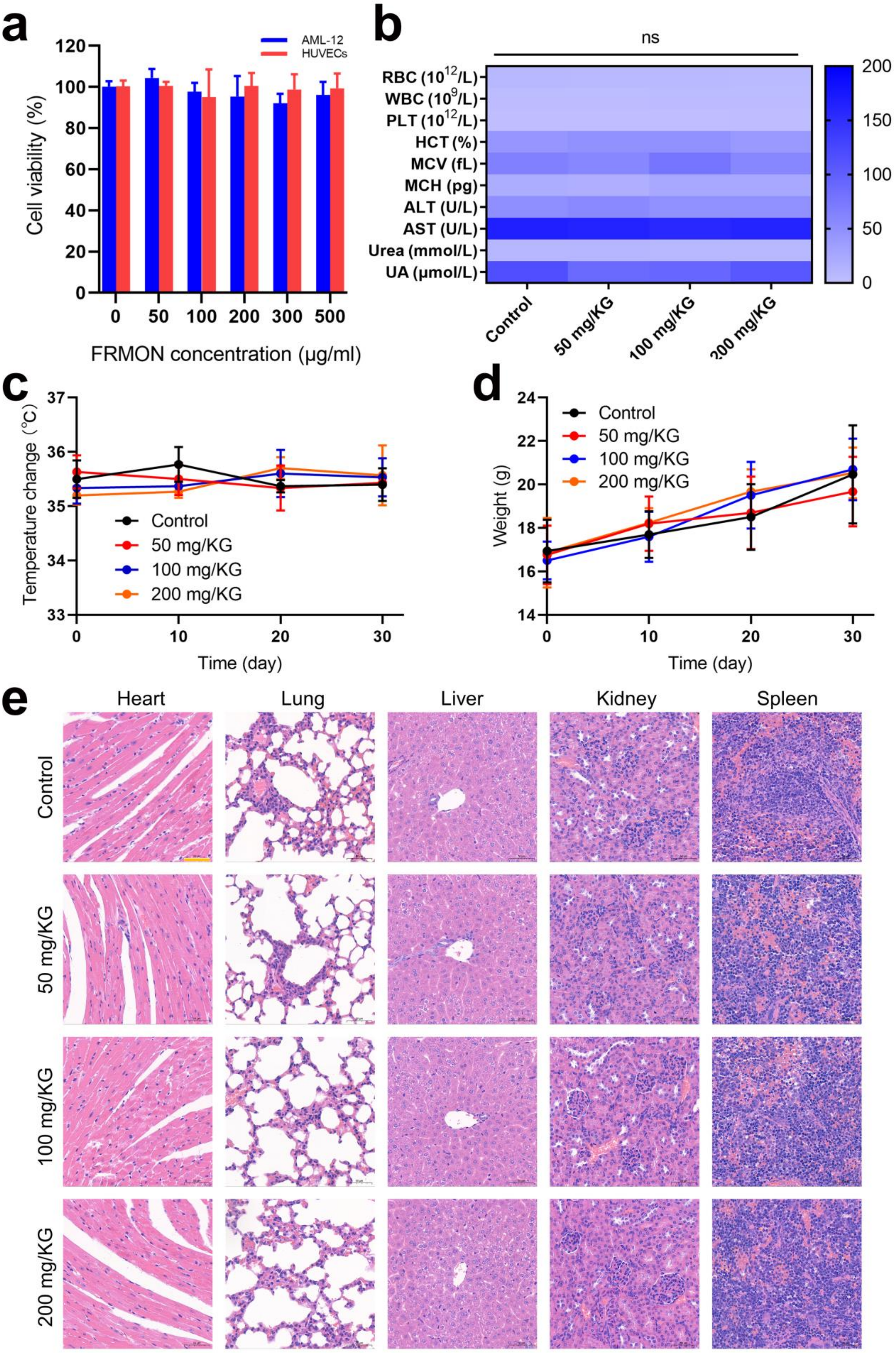

a
AML-12
HUVECs
Cell viability (%)
120
100
80
60
40
20
0
0
50
100
200
300
500
FRMON concentration (μg/ml)
b
ns
RBC (10^12/L)
WBC (10^9/L)
PLT (10^12/L)
HCT (%)
MCV (fL)
MCH (pg)
ALT (U/L)
AST (U/L)
Urea (mmol/L)
UA (μmol/L)
200
150
100
50
0
Control
50 mg/KG
100 mg/KG
200 mg/KG
c
Temperature change (°C)
37
36
35
34
33
Control
50 mg/KG
100 mg/KG
200 mg/KG
0
10
20
30
Time (day)
d
Weight (g)
24
22
20
18
16
14
Control
50 mg/KG
100 mg/KG
200 mg/KG
0
10
20
30
Time (day)
e
Heart
Lung
Liver
Kidney
Spleen
Control
50 mg/KG
100 mg/KG
200 mg/KG

Figure S25. Biosafety evaluations of FRMON *in vitro* and *in vivo*. (a) Viability of 4T1 cells after incubation with FRMON carriers as a function of concentration; (b) Heatmap showing the differences of blood and biochemical inedexes between control and FRMON. (c,d) Time-dependent temperature change (c) and weight variation (d) of mice in groups including Control and FRMON with varied concentrations. (e) Optical HE-stained microscopic images of normal organs harvested from normal mice that experienced corresponding treatment in different groups (Control and FRMON with varied concentrations) at the end of experimental period (30 days). Data are expressed as mean ± SD (n=3); and Scale bar: 50 μm..
